# Deep Learning Predicts Imminent Tumor Progression in Advanced Pancreatic Adenocarcinoma Using Serial CT Scans During Chemotherapy

**DOI:** 10.1002/mco2.70870

**Published:** 2026-07-15

**Authors:** Jun Cheng, Yize Mao, Shuxiang Huang, Xiaotong Tan, Xiaoping Yi, Xiaoying Du, Qiulin Liu, Jianyao Zhou, Rong Huang, Weijie Chen, Rong Zhang, Lizhi Liu, Wufeng Xue, Ruobing Huang, Youhui Qian, Dong Ni, Wenjun Mao, Tao Qin, Shengping Li, Qiuxia Yang

**Affiliations:** ^1^ National‐Regional Key Technology Engineering Laboratory For Medical Ultrasound Guangdong Key Laboratory For Biomedical Measurements and Ultrasound Imaging School of Biomedical Engineering Thoracic Surgery Department of the First Affiliated Hospital Shenzhen University Medical School Shenzhen University Shenzhen China; ^2^ Marshall Laboratory of Biomedical Engineering Shenzhen University Shenzhen China; ^3^ Department of Pancreatobiliary Surgery State Key Laboratory of Oncology in South China Guangdong Provincial Clinical Research Center for Cancer Sun Yat‐sen University Cancer Center Guangzhou China; ^4^ Department of Radiology Xiangya Hospital Central South University Changsha Hunan China; ^5^ National Clinical Research Center for Geriatric Disorders (Xiangya Hospital) Central South University Changsha Hunan China; ^6^ The First Affiliated Hospital of Jinan University Guangzhou China; ^7^ The First Affiliated Hospital of Bengbu Medical University Bengbu China; ^8^ Department of Radiology State Key Laboratory of Oncology in South China Guangdong Provincial Clinical Research Center for Cancer Sun Yat‐sen University Cancer Center Guangzhou China; ^9^ Department of Radiology, Affiliated Dongguan Hospital Southern Medical University Dongguan China; ^10^ School of Artificial Intelligence Shenzhen University Shenzhen China; ^11^ National Engineering Laboratory for Big Data System Computing Technology Shenzhen University Shenzhen China; ^12^ School of Biomedical Engineering and Informatics Nanjing Medical University Nanjing China; ^13^ Department of Thoracic Surgery the Affiliated Wuxi People's Hospital of Nanjing Medical University Wuxi China; ^14^ Wuxi College of Clinical Medicine Nanjing Medical University Wuxi China; ^15^ Department of Medical Oncology, Sun Yat‐sen Memorial Hospital Sun Yat‐sen University Guangzhou China

**Keywords:** deep learning, longitudinal prediction, pancreatic ductal adenocarcinoma, progressive disease, serial CT

## Abstract

Advanced pancreatic ductal adenocarcinoma (PDAC) often progresses rapidly during chemotherapy despite initial assessments of stable disease or partial response by Response Evaluation Criteria in Solid Tumors (RECIST 1.1), underscoring the limitations of the current methods for predicting short‐term progressive disease (PD). To address this, the study developed a spatiotemporal deep learning framework that integrates convolutional and long short‐term memory (LSTM) neural networks to dynamically predict PD at the next follow‐up visit using serial computed tomography (CT) scans and baseline clinical variables. The model was trained on a retrospective cohort of 243 patients (415 predicted events, defined as temporal sequences for the next follow‐up PD prediction) and evaluated across internal, external, and prospective cohorts. The model achieved area under the curve (AUC) values of 0.77, 0.76, and 0.74, respectively. Performance remained robust across chemotherapy regimens (AG or Gemcitabine‐based, FOLFIRINOX, and SOXIRI; AUC 0.68–0.79), PD subtypes (target lesion growth vs. new metastases; AUC 0.72 vs. 0.77), and baseline disease stages (locally advanced vs. metastatic; AUC 0.85 vs. 0.71). This framework enables the noninvasive, real‐time prediction of imminent PD in advanced PDAC, facilitating timely treatment modification. Its validated generalizability and reliance on routine clinical data underscore its potential for seamless integration into chemotherapy management.

## Graphical abstract

A spatiotemporal deep learning model combining serial CT scans and baseline clinical data predicts tumor progression at the next follow‐up visit in advanced PDAC. The model achieved robust performance across internal, external, and prospective cohorts (AUC: 0.74–0.77). This noninvasive approach enables earlier identification of imminent progression and supports timely treatment adjustment.

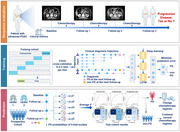

## 1 Introduction

Pancreatic ductal adenocarcinoma (PDAC) is one of the most lethal malignancies with a steadily increasing global incidence [1, 2]. Approximately 80% of patients are diagnosed at an advanced stage, including those with locally advanced or metastatic disease, with a median overall survival (OS) of approximately 12 months [3, 4, 5]. For these patients, systemic chemotherapy remains the mainstay of treatment and is typically continued until tumor progression or treatment intolerance occurs, as recommended by current practice and clinical trial protocols [6, 7, 8]. Consequently, patients with stable disease (SD) or partial response (PR) according to the Response Evaluation Criteria in Solid Tumors (RECIST 1.1) on postchemotherapy imaging are typically maintained on the same regimen. However, clinical experience shows that many of these patients develop progressive disease (PD) within a typical 2‐ to 3‐month surveillance interval. This discrepancy between imaging assessment and the subsequent clinical course highlights an urgent need for reliable tools to identify patients at risk of imminent PD, defined as disease progression within the next 2‐ to 3‐month follow‐up interval per RECIST 1.1. Such tools could enable earlier therapeutic interventions and informed treatment decisions.

Although RECIST 1.1 remains the standard for assessing chemotherapy response [9], it provides only a coarse measure of tumor burden and does not capture microstructural or biological changes that may precede radiographically measurable tumor growth [10, 11]. Radiomics and deep learning approaches have emerged as promising strategies for extracting quantitative features from medical images, allowing the recognition of subtle tumor phenotypes and spatiotemporal patterns beyond human perception [12, 13, 14, 15, 16, 17]. Previous studies, including ours, have demonstrated that image‐derived biomarkers are associated with chemotherapy responses and patient outcomes [18, 19, 20, 21, 22, 23]. However, to our knowledge, most existing studies have focused on baseline or early post‐treatment imaging to predict the initial response or long‐term survival without addressing the pressing clinical need for real‐time prediction of short‐term progression during ongoing therapy.

Current paradigms in pancreatic cancer management underscore the need for dynamic multiparameter surveillance during systemic therapy, integrating serial anatomical imaging with biological markers to guide timely therapeutic adjustments. Notably, the evolution of biological responses, assessed through longitudinal changes in imaging characteristics and serum biomarkers, such as CA19‐9, is increasingly recognized as more critical for decision‐making than static anatomical measurements alone [24]. Despite this consensus, robust tools to quantitatively decode spatiotemporal tumor heterogeneity embedded in routine serial computed tomography (CT) scans and to predict imminent progression in real time remain scarce.

Deep learning architectures that combine convolutional neural networks (CNNs) and long short‐term memory (LSTM) networks are well suited to this task. CNNs can extract spatial features that reflect tumor morphology and texture from individual CT scans [25, 26], while LSTM networks capture temporal dependencies across serial scans, modeling the longitudinal evolution of imaging features during treatment [10, 27, 28]. This synergistic architecture enables the comprehensive characterization of both intra‐scan morphology and inter‐scan progression patterns that may signal impending disease progression.

**FIGURE 1:**
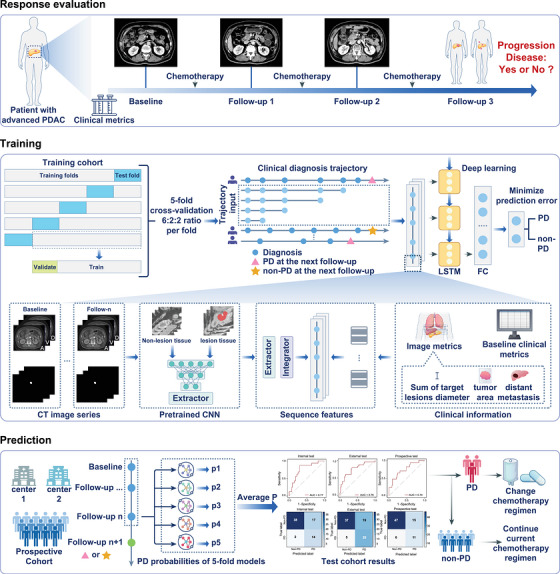
Overview of the entire research process. Serial CT scans and clinical data were collected at baseline and follow‐up visits in patients with advanced PDAC. A CNN–LSTM model was trained using longitudinal multimodal data with fivefold cross‐validation to predict PD at the next follow‐up. Model outputs from the five folds were ensembled to generate final predictions. CNN, convolutional neural network; LSTM, long short‐term memory; PD, progressive disease; PDAC, pancreatic ductal adenocarcinoma.

This multicenter study developed a CNN‐LSTM‐based model that integrates serial CT scans with clinical variables to predict short‐term tumor progression during chemotherapy in patients with PDAC (Figure 1). Model robustness was systematically evaluated across different chemotherapy regimens, progression subtypes (target lesion growth vs. new metastases), and baseline disease stages (locally advanced vs. metastatic). Model generalizability was further validated using an independent external test cohort and a prospective cohort. Collectively, these findings demonstrate the feasibility of spatiotemporal deep learning for noninvasive, real‐time prediction of disease progression, offering a potential clinical tool to guide timely therapeutic adjustments in advanced PDAC.

## 2 Results

### 2.1 Patient Characteristics

**FIGURE 2:**
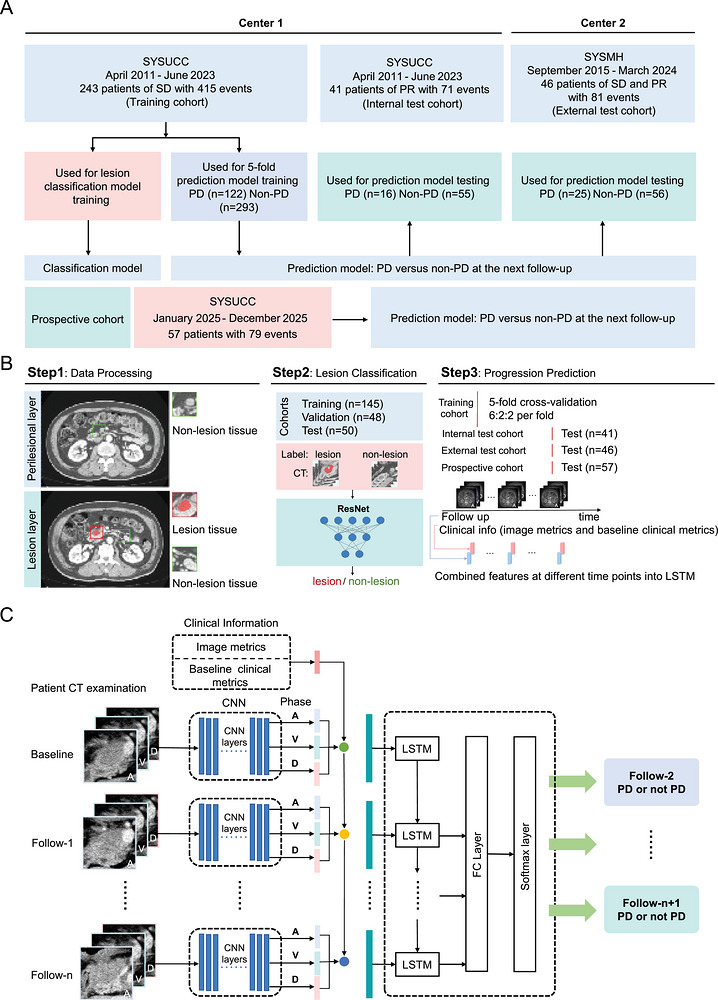
Study design flowchart. (A) Multicenter cohorts for the tumor progression prediction model: Training, internal test, external test, and prospective cohorts. (B) Flowchart of prediction model development and validation. (C) Schematic diagram of the neural network architecture for the prediction model. A, arterial phase image; D, delayed phase image; PR, Partial Response; SD, Stable Disease; SYSMH, Sun Yat‐sen Memorial Hospital; SYSUCC, Sun Yat‐sen University Cancer Center; V, venous phase image.

Figure 2A provides an overview of the retrospective and prospective patient cohorts across the centers. For the retrospective cohort, 243 patients from Center 1 (median age, 59 years; 149 men and 94 women) whose first postchemotherapy CT assessment indicated SD were enrolled as the training cohort, comprising 658 CT scans. An additional 41 patients from the same center (median age, 56 years; 21 men and 20 women) whose first assessment indicated PR constituted the internal test cohort comprising 112 CT scans. The independent external test cohort from Center 2 included 46 patients (median age, 58 years; 35 men and 11 women), of whom 42 had SD, and four had PR at the first assessment, contributing to 127 CT scans. Furthermore, a prospective cohort of 57 patients was recruited from Center 1 (median age, 59 years; 28 men and 29 women), contributing to 136 CT scans. Among these patients, 46 exhibited SD, and 11 exhibited PR at the first CT assessment. Detailed baseline characteristics of all cohorts are summarized in Table 1 and Table S1.

**TABLE 1:** Baseline demographic and clinical characteristics.

	Training cohort, *N* = 243	Internal test cohort, *N* = 41	External test cohort, *N* = 46	Prospective cohort, *N* = 57	*p* value
**Age**					0.500
Median (range), years	59 (31, 81)	56 (21, 78)	58 (37, 78)	59 (34, 79)	
**Sex**					<0.001
Female	94	20	11	29	
Male	149	21	35	28	
**Tumor location**					0.202
Head and/or neck	78	19	20	20	
Body and/or tail	165	22	26	37	
**Tumor size**					0.250
Median (range), mm	44 (18, 102)	37 (21, 97)	44.5 (20, 94)	45 (21, 92)	
**AJCC stage**					0.852
Stage III	76	14	14	21	
Stage IV	167	27	32	36	
**CA19‐9 at baseline**					0.438
Median (range), U/mL	657.6 (0.6, 33,674.0)	434.25 (2, 20,000)	591.5 (0.6, 10,000)	659.0 (2, 18,828)	
**C‐reactive protein**					0.403
Median (range), mg/L	3.705 (0.12, 109.03)	4.39 (0.2, 87.58)	4.78 (0.3, 134.75)	3.88 (0.32, 121.46)	
**Platelet count**					0.595
Median (range), 10^9^/L	219 (90, 503)	233 (116.2, 410)	202 (96, 477)	220 (95, 432)	
**Lactate dehydrogenase**					0.234
Median (range), U/L	173 (78.9, 736.7)	189 (125, 408.1)	177.5 (129, 592)	180 (130.9, 426.1)	
**Chemotherapy regimen**					<0.001
AG or gemcitabine‐based	149	19	23	17	
FOLFIRINOX	57	16	23	40	
SOXIRI	37	6	0	0	
**Prediction events**					0.330
*n*	415	71	81	79	
PD/non‐PD, *n*	122/293	16/55	25/56	17/62	

*Note: N* indicates the number of patients, and *n* indicates the number of prediction events. Multiple prediction events may arise from a single patient.

Abbreviations: AJCC, American Joint Committee on Cancer; AG, gemcitabine/nab‐paclitaxel; CA19‐9, cancer antigen 19‐9; SOXIRI, S‐1/oxaliplatin/irinotecan; FOLFIRINOX, 5‐fluorouracil/leucovorin/irinotecan/oxaliplatin.

### 2.2 Occurrence of PD Events

In the training cohort, 122 patients experienced PD, with the interval from baseline CT to PD ranging from 2.8 to 13.8 months (median: 5.4 months). Among these patients, 26 (21.3%) progressed from PR (best response before PD) to PD, whereas the remaining 96 (78.7%) progressed from SD to PD. In the internal test cohort, 16 patients experienced PD, with the time to PD ranging from 4.3 to 12.6 months (median: 6.4 months). In the external test cohort, 25 patients developed PD between 2.7 and 15.9 months after baseline (median: 5.8 months). Of these patients, seven (28.0%) progressed from PR to PD, whereas 18 (72.0%) progressed from SD to PD. In the prospective cohort, 17 patients developed PD, with time to PD ranging from 2.4 to 8.5 months (median: 5.3 months). Among these patients, five (29.4%) progressed from PR to PD, and 12 (70.6%) progressed from SD to PD.

Temporal multimodal data, including CT images, clinical variables, and CT‐derived image metrics, were used to predict PD during the next follow‐up scan. Each patient contributed multiple variable‐length event sequences, representing prediction tasks based on longitudinal data from baseline to follow‐up (e.g., baseline to follow‐up 1 and baseline to follow‐up 2). In the training cohort, 415 predicted events were generated (median number of time points per sequence, 2; range, 2–8), comprising 122 PD and 293 non‐PD events. The internal test cohort included 71 events (median time point, 2; range, 2–5), with 16 PD and 55 non‐PD events. The external test cohort comprised 81 events (median time points, 2; range, 2–5), including 25 PD and 56 non‐PD events. The prospective cohort included 79 events (median time point, 2; range, 2–5), comprising 17 PD and 62 non‐PD events.

### 2.3 Model Performance of Predicting PD at the Next Follow‐Up

**FIGURE 3:**
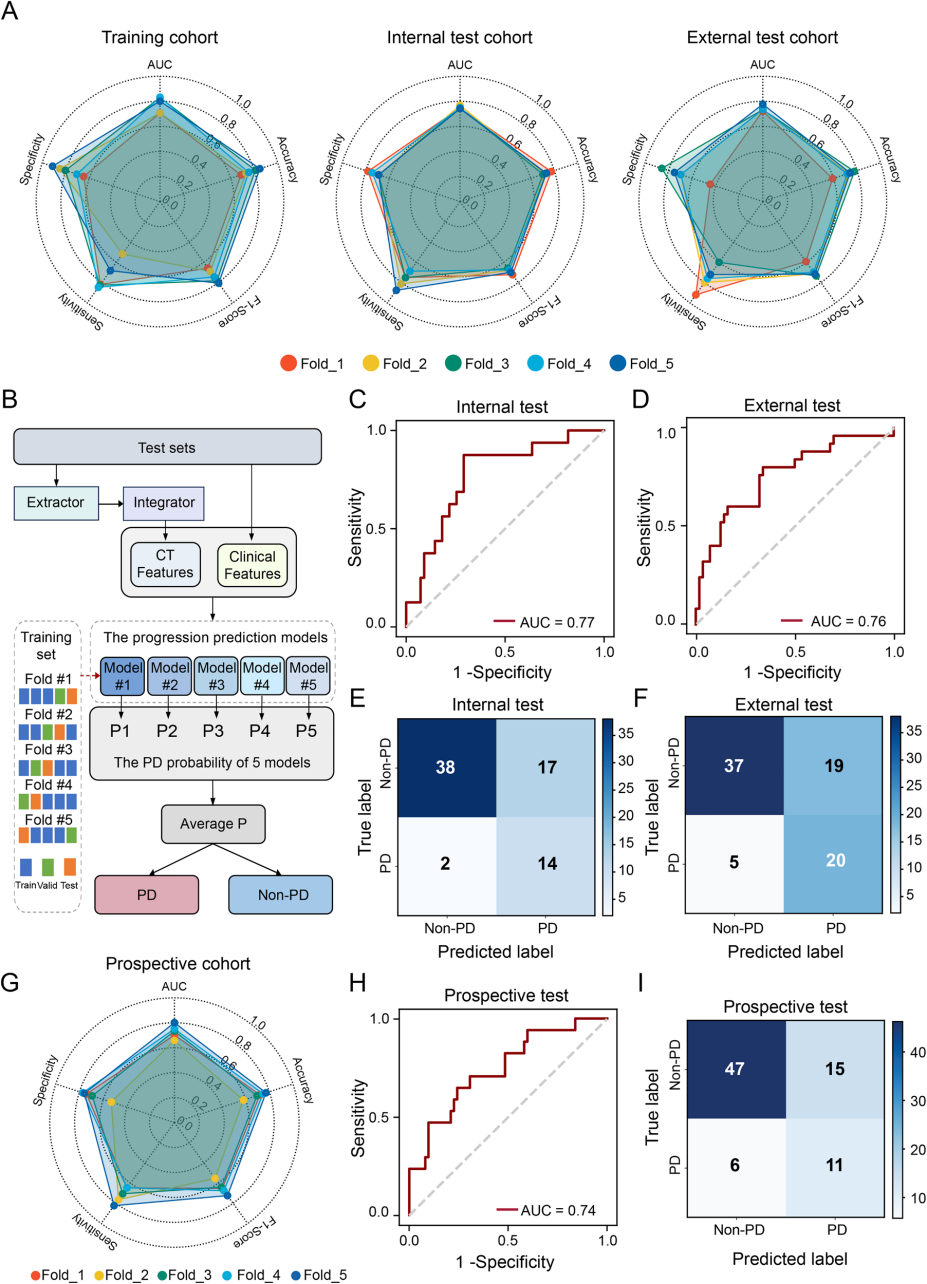
Performance of the tumor progression prediction model. (A) Performance of the fivefold cross‐validation models in the training, internal test, and external test cohorts. (B) Workflow for predicting progression in the internal and external test cohorts. Temporal multimodal features were input into five distinct models. Each output a predicted probability. The average predicted probability was used to determine the classification as PD or non‐PD. (C and D) ROC curves of the prediction model in the internal and external test cohorts. (E and F) Confusion matrices of model predictions for the internal and external test cohorts. (G–I) Performance of the fivefold models, ROC curve of the ensemble model, and confusion matrix of the ensemble model in a prospective cohort.

The prediction model was trained and tested using fivefold cross‐validation (Figure 3B). In the test subsets of the training cohort, the model demonstrated robust predictive performance, achieving a mean accuracy of 0.77 ± 0.06 (95% confidence interval [CI], 0.69–0.85). Cross‐validated receiver operating characteristic (ROC) analysis further confirmed good discriminative ability, with a mean area under the curve (AUC) of 0.78 ± 0.06 (95% CI, 0.70–0.85), with corresponding sensitivity of 0.74 ± 0.14 (95% CI, 0.57–0.91) and specificity of 0.78 ± 0.14 (95% CI, 0.65–0.91). The performance metrics of the fivefold models across the training, internal test, and external test cohorts are shown in Figure 3A and Table S2.

Model performance remained consistent in the independent internal and external test cohorts, demonstrating good generalization beyond the training data. Using the averaged predicted probabilities of the fivefold models, the model achieved AUCs of 0.77 and 0.76 in the internal and external test cohorts, respectively. The corresponding ROC curves are shown in Figure 3C,D, and confusion matrices are presented in Figure 3E,F.

**FIGURE 4:**
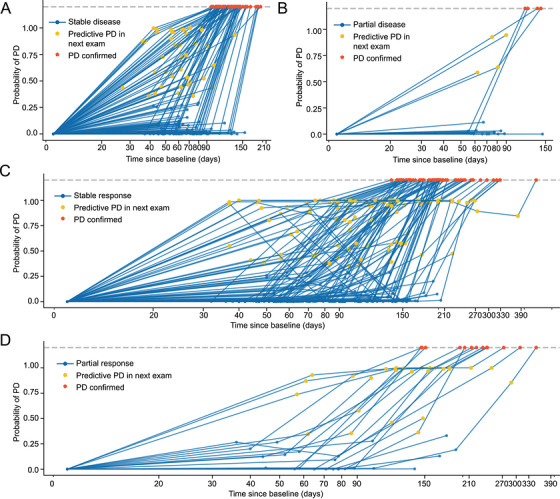
Visualization of longitudinal monitoring of predicted progression probabilities. (A) Longitudinal PD probability in the training cohort (patients with SD) with a single predictive event. Red pentagon indicates confirmed PD at threshold crossing (dashed line). (B) Longitudinal PD probability in the internal test cohort (patients with PR) with a single predictive event. Red pentagon marks confirmed PD. (C) Longitudinal PD probability in patients with SD with ≥ 2 predictive events. (D) Longitudinal PD probability in patients with PR with ≥ 2 predictive events.

Figure 4A–D illustrates the longitudinal evolution of model‐predicted PD probabilities alongside the actual PD occurrence in patients from the training and internal test cohorts at Center 1. Notably, for most patients who ultimately developed PD, the model predicted progressively increasing probabilities of PD across successive follow‐up examinations, suggesting its ability to capture early spatiotemporal signals that precede radiologically confirmed progression.

**FIGURE 5:**
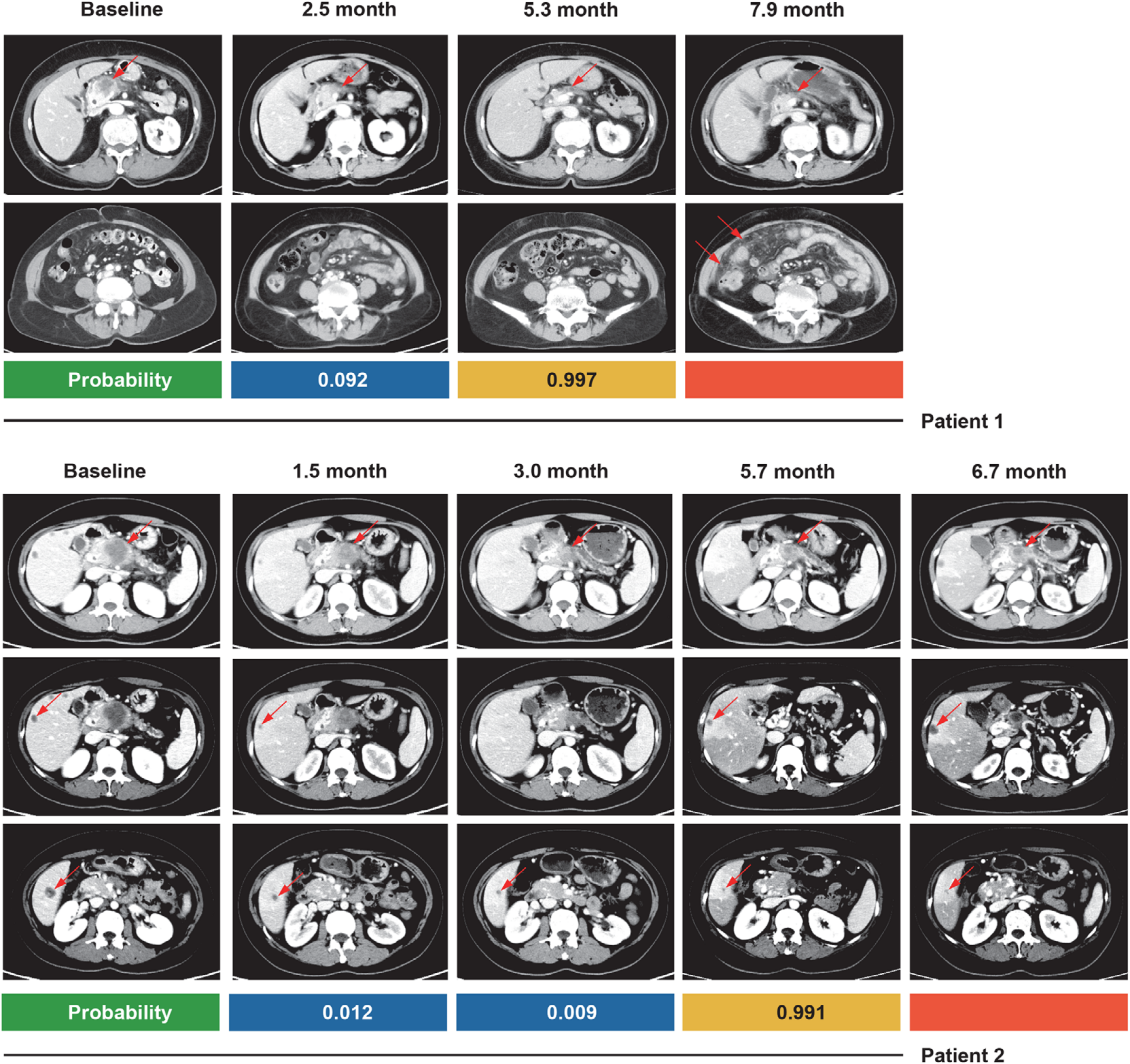
Illustration of disease progression across multiple follow‐up CT scans in two representative patients. Two representative cases from the internal test cohort are shown in which tumor progression was accurately predicted using the proposed model. Serial contrast‐enhanced CT images acquired at baseline and subsequent follow‐ups are displayed for each patient, with arrows indicating tumor lesions. The predicted probabilities of PD at each time point are shown in the corresponding images. Detailed descriptions of the imaging findings are provided in the Supporting Information.

Figure 5 presents representative longitudinal CT images from two patients in the internal test cohort, for whom disease progression was correctly predicted using the model. The predicted PD probability increased markedly at earlier follow‐up time points, preceding overt radiographic evidence of progression on subsequent CT scans, thereby illustrating the model's potential utility for early risk stratification during ongoing chemotherapy.

### 2.4 Performance of Multimodal Feature Combinations

**FIGURE 6:**
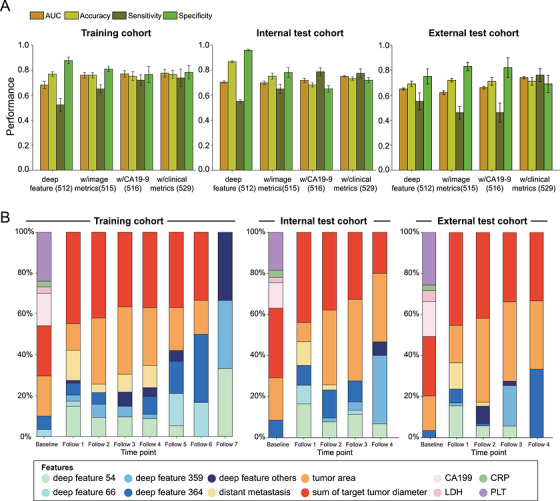
Performance comparison of models built with different feature combinations and feature contribution analyses. (A) Grouped bar charts showing the performance of different feature combinations. (B) Stacked bar charts of feature contributions across time points. X‐axis: follow‐up time‐points; Y‐axis: relative importance proportion. Color‐coded bars represent multimodal features. CRP, C‐reactive protein; LDH, lactate dehydrogenase; PLT, platelet count.

Predictive performance improved incrementally when additional features were integrated into the models (Figure 6A, Table S3). In the training cohort, the baseline model using only 512‐dimensional deep CT features achieved an AUC of 0.68. The inclusion of imaging metrics (515 features: 512 deep features plus the presence of metastases, the sum of the longest diameters of target lesions [SLD], and tumor area) and CA19‐9 levels (516 features) increased the AUC to 0.76 and 0.77, respectively. The full 529‐feature multimodal model, which further integrated hematological parameters, achieved the highest AUC of 0.78 with a balanced sensitivity (0.74) and specificity (0.78).

Similar trends were observed across the validation cohorts. In the internal test cohort, the AUC rose from 0.71 (512 features) to 0.75 (529 features). Notably, the external test cohort exhibited the most substantial gains, where the AUC improved from 0.65 (512 features) to 0.74 (529 features). These results demonstrate that expansion from single‐modality deep features to the multimodal integration of clinical and imaging variables substantially enhances both predictive performance and model generalizability.

### 2.5 Feature Contribution Analysis

The analysis of feature importance (Figure 6B) revealed distinct temporal patterns across all cohorts. At baseline in the training cohort, clinical biomarkers such as CA19‐9 and platelet count predominated the predictive contribution. Regarding CT‐derived image metrics, SLD consistently emerged as the most frequent contributor across nearly all time points, followed by the tumor area. Among the high‐dimensional deep CT features, features 54, 66, 359, and 364 were consistently identified across time points in various combinations. Notably, the relative weight of deep CT features increased progressively with longer longitudinal series. This trend suggests that temporal deep features captured from serial CT images may provide critical predictive information for impending disease progression. Similar patterns were observed across both internal and external test cohorts, further validating the robustness of the identified feature dynamics.

### 2.6 Subgroup Analyses of Model Predictive Performance

**FIGURE 7:**
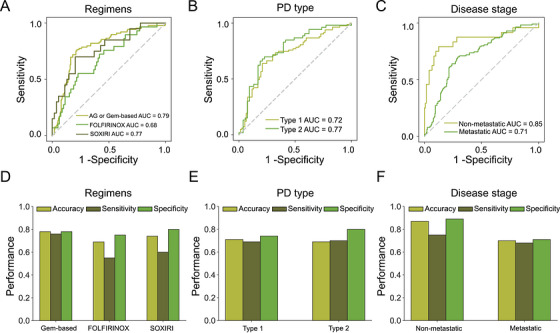
Performance of the prediction model across different clinical subgroups. (A–C) ROC curves of the prediction model in subgroups stratified by treatment regimen, progression type, and baseline disease stage. (D–F) Bar charts showing accuracy, sensitivity, and specificity of the prediction model in subgroups stratified by treatment regimen, progression type, and disease stage.

The predictive model exhibited robust generalizability across multiple clinical subgroups in the retrospective cohorts from Center 1. When stratified by treatment regimen, the model maintained stable discriminative power. In the AG or gemcitabine (GEM)‐based cohort, the model achieved an AUC of 0.79 and an accuracy of 0.78. In the FOLFIRINOX subgroup, the model yielded an AUC of 0.68 and an accuracy of 0.69. Notably, patients treated with the SOXIRI regimen showed a predictive performance with an AUC of 0.77 and an accuracy of 0.74 (Figure 7A,D, Table S4). Compared with the AG/GEM‐based reference cohort, no significant differences in AUC were observed for the FOLFIRINOX (*p* = 0.11) or SOXIRI (*p* = 0.77) subgroups.

To further assess performance by progression type, patients were stratified into type 1 PD (progression of target lesions) and type 2 PD (emergence of new metastatic lesions) subgroups. The model demonstrated comparable performance between the two subgroups, yielding AUCs of 0.72 for type 1 and 0.77 for type 2 (Figure 7B,E; *p* = 0.51). The corresponding accuracies were 0.71 and 0.74, respectively (Table S5). These findings indicate that the model effectively captures distinct biological manifestations of disease progression.

Performance was assessed based on the baseline disease stage. The model showed superior predictive power in patients with non‐metastatic (locally advanced) disease compared with those with metastatic disease (AUC 0.85 vs. 0.71, accuracy 0.87 vs. 0.70; *p* = 0.0363) (Figure 7C,F, Table S6). This performance gap likely reflects the heightened tumor heterogeneity and biological complexity inherent in metastatic states.

Subgroup analysis by tumor location showed relatively consistent performance between head/neck and body/tail tumors. The model achieved AUCs of 0.73 and 0.78 (*p* = 0.38) and accuracies of 0.75 and 0.75, respectively (Table S7). The sensitivity was lower in the head/neck subgroup (0.61 vs. 0.73), whereas the specificity was slightly higher (0.80 vs. 0.76). Overall, the model maintained acceptable predictive performance in both subgroups, suggesting its robustness across anatomical locations.

Performance was also evaluated according to the follow‐up sequence length. Although overall AUC values were similar between the short‐sequence (≤ 2 follow‐up events) and long‐sequence (> 2 follow‐up events) groups (0.73 vs. 0.71; *p* = 0.91), the long‐sequence subgroup demonstrated higher sensitivity (0.97 vs. 0.62) and accuracy (0.89 vs. 0.72), accompanied by lower specificity (0.57 vs. 0.79) (Table S8). These findings suggest that a longer longitudinal follow‐up period may provide additional temporal information to improve the sensitivity of progression prediction.

### 2.7 Comparison With Other Methods

The proposed LSTM‐based method demonstrated superior, more robust performance across all cohorts compared with alternative approaches (Table S9). Traditional machine learning models (support vector machine [SVM] and logistic regression) using single‐time‐point data achieved limited predictive power, with AUCs ranging from 0.49 to 0.62 across the training, internal test, and external test sets. These models exhibit a significant bias toward negative predictions (non‐PD), characterized by high specificity but low sensitivity.

The transformer‐based model yielded an improved performance in the training (AUC = 0.68), internal test (AUC = 0.79), and external test (AUC = 0.67) sets. Ablation studies confirmed that incorporating temporal dependencies through the time‐positional encoding (TPE) and time‐sensitive multihead self‐attention (TMSA) modules enhanced model performance in the training and internal test sets (Table S10).

To further evaluate the clinical applicability, decision curve analysis was performed in patients with elevated baseline CA19‐9 levels (CA19‐9 > 35 U/mL) from the entire retrospective cohort at Center 1 (*n* = 262). The proposed model was compared to a longitudinal CA19‐9‐based model for predicting PD. As shown in Figure S1, across a wide range of threshold probabilities (0.137–0.600), this model consistently yields a higher net benefit than both the CA19‐9‐based strategy and the default treat‐all or treat‐none strategies. In contrast, CA19‐9 demonstrated limited discriminative performance (AUC = 0.58), and its net benefit declined rapidly as the probability threshold increased, indicating limited clinical utility in guiding treatment decisions.

### 2.8 Further Validation on a Prospective Cohort

To further evaluate the clinical utility and robustness of the proposed model, an independent validation was conducted using a prospective cohort. Model performance was first assessed using the five models derived from fivefold cross‐validation on the training cohort, which demonstrated stable performance across multiple metrics (Figure 3G). By averaging their predicted probabilities, an ensemble model achieved an AUC of 0.74 (Figure 3H), confirming its reliable discriminative power for predicting disease progression in a real‐world clinical setting. The corresponding confusion matrix provided a detailed breakdown of the classification results (Figure 3I). Of the 79 predicted events, the model correctly identified 47 true non‐PD and 11 true PD events, whereas 15 non‐PD and six PD events were misclassified.

## 3 Discussion

This study presents a CNN‐LSTM deep learning model that dynamically predicts short‐term PD in patients with advanced PDAC undergoing chemotherapy. By integrating serial CT scans with clinical features, this model captures spatiotemporal tumor evolution and provides a real‐time, noninvasive prediction of imminent progression. This approach enables individualized treatment adjustments based on evolving multimodal features, thereby addressing a critical unmet need in clinical oncology.

Serial imaging surveillance is essential in monitoring treatment response and tumor evolution. This study revealed that a substantial proportion of patients initially assessed as having SD or PR during chemotherapy progressed rapidly to PD within a short interval. Approximately 75% of PD events originated from the SD status, whereas 25% arose from the PR status, consistent with previous findings [18]. The proposed model accurately identifies PD risk at the next scheduled imaging assessment, outperforming the traditional RECIST 1.1 criteria by flagging high‐risk patients before radiological progression becomes evident. This capability provides clinicians with a crucial therapeutic window for a timely intervention.

Notably, the model demonstrated stable performance across multiple validation cohorts. It was trained on patients with SD, tested internally on a PR‐only cohort, externally tested on a mixed SD/PR cohort, and further validated on a prospective cohort with AUCs ranging from 0.74 to 0.77. Its robust predictive power across subgroups, including chemotherapy regimens, PD subtypes, and baseline disease stages, underscores its strong generalizability across diverse clinical scenarios. As the model relies solely on routine clinical inputs (serial CT scans, RECIST 1.1‐based measurements, and baseline hematological tests), it holds considerable promise for seamless integration into chemotherapy workflows as a decision‐support tool at minimal or no additional cost.

This robust performance indicates that the model offers a practical technical solution to a key challenge in advanced pancreatic cancer management: the need for a dynamic, multimodal assessment of response. Recent consensus highlights that tumor biology evolution, not just anatomy, dictates outcomes, and calls for tools that integrate serial imaging with biomarkers for real‐time decision support [24]. By quantifying spatiotemporal tumor heterogeneity from routine CT scans, the CNN‐LSTM framework generated a digital longitudinal biomarker that predicts progression earlier than RECIST, thereby enabling timely, biology‐informed treatment adjustments.

Methodologically, this study employed a CNN‐LSTM architecture to model temporal multimodal data. The CNN component was pretrained to extract higher order CT features [29], capturing nuanced morphological and textural changes beyond simple lesion size. Specific deep features (e.g., 54, 66, 359, and 364) consistently emerged as core predictors. The LSTM network further analyzed longitudinal dependencies and dynamically tracked the evolution of tumor heterogeneity. Feature contribution analysis revealed that the predictive weight of recent deep CT features increased over time, indicating that immediate tumor biological states carry greater prognostic value than baseline or historical data. This finding validates the LSTM's ability to capture dynamic tumor heterogeneity and supports the importance of sequential imaging analysis.

Similar trends have been observed for other cancers. For instance, Xu et al. demonstrated that incorporating serial CT scans improved lung cancer survival prediction compared with a single pre‐treatment CT scan [28], highlighting the critical role of longitudinal morphological changes. Similarly, He et al. reported that time‐series imaging significantly improved OS stratification in stage IV gastric cancer compared with baseline or single‐follow‐up models [30], confirming that temporal heterogeneity evolution optimizes prognosis prediction. The CNN‐LSTM framework quantifies dynamic morphological changes during chemotherapy, overcoming the limitations of static imaging models and enabling the early detection of subclinical tumor progression. While previous studies have demonstrated LSTM's utility in predicting treatment response in lung cancer [28], metastatic colorectal cancer [10], and prostate cancer [27] using longitudinal CT or MRI images, to our knowledge, this study represents the first application of LSTM to dynamic chemotherapy response prediction in PDAC.

Model comparisons confirmed that integrating deep CT features with image metrics and baseline clinical parameters enhanced predictive performance, demonstrating the synergistic benefits of multimodal data [31, 32]. Whole‐tumor CT deep features systematically encoded the morphological heterogeneity and biological characteristics of pancreatic tumors, whereas conventional metrics such as SLD provided complementary information on the total target lesion burden. The presence of distant metastasis predicts outcome, a known prognostic factor in tumor, node, and metastasis (TNM) stage IV disease [33], and contributes to prediction, corroborating previous studies [34] linking metastasis with reduced progression‐free survival (PFS) and treatment response. Among the baseline laboratory features, the CA19‐9 level and platelet count were key predictors. High baseline CA19‐9 levels predict poor survival in borderline resectable PDAC [35, 36], and their decline during chemotherapy [37, 38] and normalization post‐neoadjuvant therapy [39] indicate favorable outcomes in advanced PDAC. Patients with PDAC often exhibit altered platelets [40]. Elevated platelet count is associated with poor survival in many cancer types [41, 42]. Previous ensemble models that combine preoperative clinical data (including CA19‐9 and platelet count) with deep learning‐based CT features have also demonstrated good post‐surgery survival prediction in patients with PDAC [31]. The selection of readily available baseline parameters enhances the model's clinical applicability. Future prospective studies should explore the value of monitoring the dynamic biomarkers during chemotherapy.

This study had several limitations. First, the study design may have introduced selection bias, as inclusion required serial imaging follow‐up (≥ 3 CT scans), potentially underestimating real‐world heterogeneity due to interrupted follow‐up or missing imaging data. Second, although the external and prospective validations confirmed the model's generalizability, the cohort size was relatively small, partly due to stringent inclusion criteria that required serial regular CT follow‐up. Therefore, larger multicenter studies are warranted. Third, while the feature fusion strategy enhanced predictive performance, the interpretability of deep features remained limited by the inherent “black‐box” nature of deep learning, although feature contribution analysis partially elucidated key predictors. Linking imaging features to biological tumor mechanisms, such as correlations with histopathological findings, genomic alterations, or treatment resistance pathways, may improve clinical interpretability and mechanistic understanding. Fourth, manual tumor segmentation was performed. Integrating CNN‐based automated segmentation algorithms can improve scalability and clinical utility in future implementations. Finally, effective second‐line treatment options for advanced PDAC remain limited, undermining the immediate clinical benefits of early prediction. Nevertheless, this model provides valuable risk stratification, particularly for patients with high tumor burden, and timely intervention may help prevent disease progression. However, for patients who tolerate the current treatment and have a low tumor burden, the model's predictions should be interpreted with caution, and any decision to change the chemotherapy regimen should be guided by clinical judgment.

In conclusion, the proposed deep learning network, which leverages serial CT images and baseline clinical data, effectively provides a dynamic prediction of PD during chemotherapy for advanced PDAC, independent of diverse clinical scenarios (such as chemotherapy regimens and baseline disease stage). This model enables early identification of high‐risk patients, serving as a clinically valuable complement to RECIST 1.1 criteria and creating a therapeutic decision window for personalized treatment adjustment. Future research should focus on large multicenter validation and explore associations between deep imaging features and tumor biological mechanisms to facilitate clinical translation, ultimately optimizing the precision management of advanced PDAC.

## 4 Materials and Methods

### 4.1 Patients and Study Design

This study included patients from two tertiary academic medical centers (Sun Yat‐sen University Cancer Center, Center 1, and Sun Yat‐sen Memorial Hospital, Center 2). An overview of the research process is presented in Figure 1.

Consecutive patients with advanced PDAC who received chemotherapy at Center 1 between April 2011 and June 2023 were retrospectively reviewed. The inclusion criteria were as follows: (1) pathologically confirmed advanced PDAC; (2) receiving first‐line chemotherapy at Center 1 with ≥ 3 treatment cycles [18, 43]; (3) initial post‐chemotherapy efficacy evaluation showing SD or PR per RECIST 1.1 criteria; (4) availability of multiple triple‐phase enhanced CT scans (≥ 3 times), with 1.5‐ to 3‐month intervals between consecutive scans; and (5) complete clinical treatment documentation. The detailed exclusion criteria are presented in the Supporting Information, and the patient selection flowchart is shown in Figure S2. Eligible patients from Center 1 were divided into a training cohort (patients with SD) and an internal test cohort (patients with PR) (Figure 2A). Similarly, eligible consecutive patients with SD and PR from Center 2 between September 2015 and March 2024 were enrolled as independent external test cohorts. Additionally, a prospective cohort was recruited from Center 1 between January 2025 and December 2025.

Clinical data, including demographic characteristics (age and sex), baseline hematological parameters (CA19‐9 levels, complete blood count, and serum biochemistry), tumor location, TNM stage, American Joint Committee on Cancer (AJCC) stage, and chemotherapy regimen, were obtained from medical records.

### 4.2 Chemotherapy Treatment and Response Evaluation

All patients received standard chemotherapy after diagnosis. The regimens included AG (GEM/nab‐paclitaxel) or GEM‐based combinations, FOLFIRINOX (5‐fluorouracil/leucovorin/irinotecan/oxaliplatin), and S‐1/oxaliplatin/irinotecan (SOXIRI). Follow‐up CT examinations were conducted every 2–3 months during chemotherapy.

The study endpoint was defined as the time of discontinuation of first‐line chemotherapy due to disease progression or patient‐related factors (e.g., intolerance), initiation of radiotherapy, or surgical resection/nanoknife ablation (typically following a favorable treatment response).

Details of the CT acquisition are described in the Supporting Information. Tumor response was assessed using serial post‐chemotherapy follow‐up CT scans according to RECIST 1.1 criteria [9]. Two experienced radiologists (one with > 10 years of subspecialty experience in pancreatic oncology and another with 5 years of experience) independently reviewed all scans. The readers were blinded to the clinical data, including the treatment regimens and outcomes. Any discrepancies were resolved by consensus. At each time point, both radiologists independently measured the longest diameter of the target lesions from baseline to the study endpoint. The reported values represent the means of both measurement sets, and the SLD was calculated for each evaluation.

### 4.3 Feature Extraction and Integration

Volumes of interest encompassing the entire pancreatic tumor (5‐mm slice thickness) were manually contoured on the arterial, venous, and delayed‐phase CT images. Details of the tumor segmentation and image preprocessing are described in the Supporting Information.

Deep image features were extracted using a CNN based on the ResNet‐50 architecture, initialized with ImageNet pre‐trained weights, and applied to 2D tumor slices from the three CT phases (Figure 2B,C). To ensure informative feature extraction from lesions, a pancreatic tumor recognition model was trained using all the CT images in the training set (including baseline and follow‐up scans). This model distinguishes tumor tissue from surrounding normal tissue, enabling reliable extraction of lesion‐specific features. The training details are provided in the Supporting Information. The best‐performing CNN model (accuracy: 0.881; AUC, 0.954) was selected as the final feature extractor, generating a 512‐dimensional feature vector from the last convolutional layer for each 2D slice. Gradient‐weighted class activation mapping (Grad‐CAM) analysis showed that the model's attention was primarily concentrated within the annotated tumor regions (Figure S3).

For a given time point, the slice‐level features from each phase were aggregated using a tumor‐area‐weighted approach. Within each phase, the feature vectors of all slices were combined and weighted by their relative tumor cross‐sectional areas to produce a single 512‐dimensional phase‐specific feature. The phase‐specific features from the arterial, venous, and delayed phases were then summed to yield a final 512‐dimensional deep feature representation at each time point. Direct summation was selected over concatenation or attention‐based fusion to preserve fixed feature dimensionality and improve robustness to potentially incomplete multiphasic CT acquisitions. Furthermore, this approach avoids introducing additional fusion‐specific trainable parameters and reduces overall model complexity.

In addition to deep imaging features, this study incorporated baseline clinical variables (including age, sex, CA19‐9 levels, complete blood count, and serum biochemistry parameters) and CT‐derived image metrics at each time point. The image metrics included the presence of metastases and SLD, as well as the maximum cross‐sectional tumor area (defined as the number of tumor pixels in the largest axial slice). Because follow‐up hematological data were incomplete, only the baseline hematological parameters were used. The baseline feature set comprised 17 dimensions (clinical variables and imaging metrics). For subsequent time points, missing clinical variables were zero‐padded to ensure consistent feature dimensionality across the time points. Compared with forward‐fill imputation (i.e., carrying the last available clinical measurement to fill subsequent missing time points), this approach avoids propagating previous laboratory measurements to later time points and shows a slightly better empirical performance (AUC 0.78 vs. 0.76).

Finally, the 512‐dimensional deep CT features were concatenated with 17‐dimensional clinical and imaging metrics, yielding a 529‐dimensional multimodal feature vector at each time point. This study additionally evaluated a Feature‐wise Linear Modulation (FiLM) module [44] as an alternative fusion method; however, it did not improve performance in cross‐validation experiments, and a straightforward concatenation strategy was therefore adopted as the final model. To preserve the native distribution of the clinical biomarkers, the original feature scales were retained before multimodal concatenation (comparative feature‐scaling experiments are detailed in Table S11). This multimodal vector was used as the input for subsequent LSTM‐based temporal modeling.

### 4.4 Development of an LSTM‐Based Prediction Model

In this study, a longitudinal event was defined as a time series spanning the patient's baseline CT scan to any subsequent follow‐up CT scan. Each event incorporated temporal multimodal data from at least two time points, with the event label indicating whether PD occurred at the next follow‐up scan (Tables S12–S15). Under this framework, a patient with *n* + 1 CT scans contributed *n* − 1 event sequences of at least two time points (i.e., *n* − 1 variable‐length time series). If PD was observed during the study period, the final event was labeled 1 (PD occurred), whereas all preceding events were labeled 0 (PD did not occur). If no PD was observed, all event labels were assigned a value of 0.

LSTM networks were employed to model temporal dependencies in multimodal feature sequences (Figure 2B,C). Through its gating mechanisms, the LSTM captures evolving patterns across time points and produces a 256‐dimensional hidden state vector after multilayer stacking. This vector was then passed through a fully connected layer and a SoftMax classification layer to predict the probability of PD occurrence.

For both the CNN feature extraction and LSTM temporal modeling stages, categorical cross‐entropy was used as the loss function, and the Adam optimizer was used for model training. The detailed experimental settings and computational environments are provided in the Supporting Information.

### 4.5 Fivefold Cross‐Validation and Multicenter Validation

A prediction model was developed and evaluated using fivefold cross‐validation within the training cohort to optimize the hyperparameters for each fold (Figure 2B). The cohort was partitioned into five patient‐level subsets, and all prediction events from the same patient were assigned to the same fold to prevent data leakage across datasets. In each fold, 60% of the patients were used for training, 20% for validation (hyperparameter tuning), and the remaining 20% for testing the corresponding model.

Subsequently, an ensemble prediction was generated by averaging the probability outputs of all five models to determine PD status. This ensemble model was then rigorously evaluated using independent internal tests, external tests, and prospective cohorts to assess the generalization performance (Figure 3B). The optimal threshold for binary classification (0.338) was determined using the Youden index [45] for the training cohort.

### 4.6 Feature Contribution Analysis

To elucidate the model's predictive basis, an Integrated Gradient algorithm from the Captum library (https://captum.ai/) was employed to analyze feature contributions. For each time point, the top 10 contributing features were selected from the 512‐dimensional CT deep features. Subsequently, these were combined with 17 clinical/imaging features to identify the top three highest contributing features at each time point. This process reduces the original high‐dimensional time‐series data (number of time points × 529 features) to a compact set of key features (number of time points × 3 features). The frequency of these high‐contribution features was systematically quantified across the fivefold cross‐validation test subsets, the internal test cohort, and the external test cohort. Finally, the top five most frequent high‐contribution features were identified and visualized with bar plots.

### 4.7 Subgroup Analyses

To evaluate model performance across subgroups, patients were stratified by five clinical variables: chemotherapy regimen, PD type, baseline disease stage, tumor location, and follow‐up sequence length. First, regarding the chemotherapy regimen, all patients in the retrospective cohort from Center 1 were stratified into three subgroups: AG or GEM‐based, FOLFIRINOX, and SOXIRI. Second, considering that event labels (PD occurrence) were defined per RECIST 1.1, patients with PD occurrence were further divided into two progression categories: type 1, target lesion increase meeting the PD criteria (pancreatic lesion enlargement, metastatic enlargement, or synchronous enlargement) with or without new metastases or non‐target lesion progression; and type 2, new lesions exclusively or isolated non‐target lesion progression meeting the PD criteria. Furthermore, patients were stratified according to baseline disease stage (locally advanced vs. metastatic), tumor location (head/neck vs. body/tail), and follow‐up sequence length (≤ 2 vs. > 2 follow‐up events).

### 4.8 Comparison With Other Methods

#### 4.8.1 Prediction Using Single‐Time‐Point Data

To validate the benefits of using temporal sequence information, a comparative experiment was conducted using single‐time‐point data. The feature‐extraction procedure was identical to that used in the main method. For each follow‐up CT scan, 512‐dimensional deep features were extracted using a pretrained feature extractor. These deep features were concatenated with imaging‐derived metrics and baseline clinical variables. The resulting 529‐dimensional feature vector served as the input for the machine learning models, including SVM and logistic regression. The same fivefold cross‐validation and multicenter validation procedures as previously described were employed to ensure a fair comparison.

#### 4.8.2 Transformer‐Based Method

Figure S4 illustrates the proposed transformer‐based deep learning framework for predicting disease progression in pancreatic cancer. The encoder uses 512‐dimensional deep image features extracted from CT scans as input, whereas the decoder incorporates both imaging‐derived metrics and clinical variables. To better model the sequential follow‐up data, a TPE mechanism and a TMSA module were introduced to enhance the transformer's ability to capture temporal dependencies. TPE jointly encodes the relative temporal order and actual time intervals between follow‐up scans. TMSA enables the model to assign greater attention to time points closer to the prediction target, reflecting their stronger clinical relevance. Detailed descriptions are provided in the Supporting Information.

#### 4.8.3 Prediction Based on the Delta of CA19‐9 Between Two Follow‐Ups

To benchmark the clinical utility of CA19‐9, a separate analysis was conducted using longitudinal CA19‐9 measurements from the retrospective cohort at Center 1. To ensure a biologically meaningful comparison, patients with normal baseline CA19‐9 levels (≤ 35 U/mL) were excluded because they may be Lewis antigen‐negative and unable to produce CA19‐9 or Lewis antigen‐positive cells with intrinsically low CA19‐9 expression. Therefore, only patients with elevated baseline CA19‐9 levels (CA19‐9 > 35 U/mL) in the training and internal testing cohorts were included (*n* = 262). For each follow‐up, the change in CA19‐9 levels relative to the previous visit was calculated and used as input to a random forest model to predict PD at the subsequent follow‐up. Model training was performed using fivefold cross‐validation in the training cohort, followed by independent evaluation in the internal test cohort.

### 4.9 Statistical Analyses

Continuous variables are reported as medians and ranges, while categorical variables are reported as frequencies and percentages. Comparisons of baseline characteristics across cohorts were performed using the Kruskal–Wallis test for continuous variables and the chi‐square test or Fisher's exact test for categorical variables, as appropriate. Interobserver reproducibility was assessed using the Dice similarity coefficient (DSC), intraclass correlation coefficient (ICC), and Cohen's kappa. The model performance was evaluated using ROC curves and the corresponding AUC values. The accuracy, sensitivity, specificity, and F1‐score have also been reported. The DeLong test was used to compare the AUCs across clinical subgroups. Decision curve analysis was used to assess and compare the clinical utility of the proposed model and CA19‐9 levels. All statistical analyses were performed using the R software (version 4.4.1; R Foundation for Statistical Computing) within the RStudio environment (version 2022.7.1.554).

## Author Contributions

Y.M., J.C., and Q.Y. supervised the study. Q.Y. managed ethics approvals. J.C., Y.M., S.H., X.T., and Q.Y. conceived and designed the study and performed the formal analysis. X.D., W.X., R.H., Y.Q., and X.Y. supervised the methodology and verified analytical accuracy. Q.L., J.Z., R.H., W.C., and T.Q. collected the multicenter data. J.C., Y.M., Q.Y., S.H., and X.T. drafted the initial manuscript. R.Z., L.L., D.N., and W.M. critically revised the manuscript. J.C., Y.M., T.Q., and S.L. were responsible for visualization, investigation, and supervision. All authors reviewed and approved the final version before submission.

## Funding

This work was supported by Guangdong Basic and Applied Basic Research Foundation (Nos. 2025A1515011821 and 2026A1515012488 to J.C.), Shenzhen Science and Technology Program (No. JCYJ20240813143302004 to J.C.), 2024 Hospital‐Level Clinical Research Key Project (20243357014 to Y.Q.), Guangzhou Science and Technology Innovation Fund (No. 2024P‐GX19 to S.L.), National Natural Science Foundation of China (Nos. 12326619 and 62171290 to D.N.), Science and Technology Planning Project of Guangdong Province (No. 2023A0505020002 to D.N.), and Frontier Technology Development Program of Jiangsu Province (No. BF2024078 to D.N.).

## Ethics Statement

The Institutional Review Board of Sun Yat‐Sen University Cancer Center approved the study (approval number: B2022‐748 for the retrospective cohorts, B2025‐023 for the prospective cohort). All procedures were performed in accordance with the Declaration of Helsinki. Written informed consent was waived on the grounds that only de‐identified, routinely acquired imaging and clinical data were analyzed. However, written informed consent was obtained from all patients prior to their treatment and imaging examinations.

## Conflicts of Interest

The authors declare no conflicts of interest.

## Supporting information



Supporting Information:Mco270870‐sup‐0001‐SuppMat.docx

## Data Availability

The datasets generated and/or analyzed during the current study are not publicly available, but are available from the corresponding author on reasonable request. All original code has been deposited at https://github.com/HSussane/Pancreas_Progress_Prediction. Any additional information required to reanalyze the data reported in this paper is available from the lead contact (yangqx@sysucc.org.cn) upon request.
