# Supplementary material for: Deep Learning Predicts Imminent Tumor Progression in Advanced Pancreatic Adenocarcinoma Using Serial CT Scans During Chemotherapy
Source: MedComm (2020). 2026 Jul 15;7(8):e70870. doi: 10.1002/mco2.70870 (PMC13370104; doi:10.1002/mco2.70870)
Supplement: Supplementary file 1 — Supporting Information:Mco270870‐sup‐0001‐SuppMat.docx [file MCO2-7-e70870-s001.docx]

**Supplemental Material**

**Deep learning predicts imminent tumor progression in advanced pancreatic adenocarcinoma using serial CT scans during chemotherapy**

**Authors**

**Jun Cheng**^1,2 #^**, Yize Mao**^3 #^**, Shuxiang Huang**^1,2 #^**, Xiaotong Tan**^1,2 #^, **Xiaoping Yi**^4,5#^, Xiaoying Du^6,7^, Qiulin Liu^8^, Jianyao Zhou^9^, Rong Huang^8^, Weijie Chen^8^, Rong Zhang^8^, Lizhi Liu^8^, Wufeng Xue^1,2^, Ruobing Huang^1^, Youhui Qian^1^, Dong Ni^2,10,11,12^, **Wenjun Mao**^13,14*^**, Tao Qin**^15*^**, Shengping Li**^3*^**, Qiuxia Yang**^8*^

^#^These authors contributed equally.

**Affiliations**

1. National-Regional Key Technology Engineering Laboratory for Medical Ultrasound, Guangdong Key Laboratory for Biomedical Measurements and Ultrasound Imaging, School of Biomedical Engineering, Thoracic Surgery Department of the First Affiliated Hospital, Shenzhen University Medical School, Shenzhen University, Shenzhen 518055, China

2. Marshall Laboratory of Biomedical Engineering, Shenzhen University, Shenzhen 518055, China

3. Department of Pancreatobiliary Surgery, State Key Laboratory of Oncology in South China, Guangdong Provincial Clinical Research Center for Cancer, Sun Yat-sen University Cancer Center, Guangzhou 510060, China

4. Department of Radiology, Xiangya Hospital, Central South University, 410008 Changsha, Hunan, China

5. National Clinical Research Center for Geriatric Disorders (Xiangya Hospital), Central South University, 410008 Changsha, Hunan, China

6. The First Affiliated Hospital of Jinan University, Guangzhou 510632, China

7. The First Affiliated Hospital of Bengbu Medical University, Bengbu 233000, China

8. Department of Radiology, State Key Laboratory of Oncology in South China, Guangdong Provincial Clinical Research Center for Cancer, Sun Yat-sen University Cancer Center, Guangzhou 510060, China

9. Department of Radiology, Affiliated Dongguan Hospital, Southern Medical University, Dongguan, China

10. School of Artificial Intelligence, Shenzhen University, Shenzhen 518060, China

11. National Engineering Laboratory for Big Data System Computing Technology,

Shenzhen University, Shenzhen 518060, China

12. School of Biomedical Engineering and Informatics, Nanjing Medical University,

Nanjing 211166, China

13. Department of Thoracic Surgery, the Affiliated Wuxi People’s Hospital of Nanjing Medical University, Wuxi 214023, China.

14. Wuxi College of Clinical Medicine, Nanjing Medical University, Wuxi 214023, China

15. Department of Medical Oncology, Sun Yat-sen Memorial Hospital, Sun Yat-sen University, Guangzhou, China

**Correspondence**

**Qiuxia Yang,** Department of Radiology, State Key Laboratory of Oncology in South China, Guangdong Provincial Clinical Research Center for Cancer, Sun Yat-sen University Cancer Center, Guangzhou 510060, China. Email: yangqx@sysucc.org.cn

**Shengping Li,** Department of Pancreatobiliary Surgery, State Key Laboratory of Oncology in South China, Guangdong Provincial Clinical Research Center for Cancer, Sun Yat-sen University Cancer Center, Guangzhou 510060, China. Email: lishp@sysucc.org.cn

**Tao Qin,** Department of Medical Oncology, Sun Yat-sen Memorial Hospital, Sun Yat-sen University, Guangzhou, China. Email: qint6@mail.sysu.edu.cn

**Wenjun Mao,** Department of Thoracic Surgery, the Affiliated Wuxi People’s Hospital of Nanjing Medical University, Wuxi 214023, China. Email: maowenjun1@njmu.edu.cn

**Contents**

**Supplementary Methods**

Exclusion criteria

CT image acquisition

Evaluation of inter-observer agreement

Volume-of-interest segmentation

CT image preprocessing

Deep feature extraction for pancreatic tumor

Comparison with other methods

Experimental setup

References

**Supplementary Tables**

Supplementary Tables 1-15

**Supplementary legend for Figure 5**

**Supplementary Figures**

Supplementary Figures 1-4

# Supplementary Methods

## Exclusion criteria

The following patients were excluded:

1. Patients without a first efficacy evaluation: (a) patients who received prior treatment in another hospital or received incomplete first-line chemotherapy at our center; (b) patients without baseline imaging data at our center; and (c) patients without any follow-up imaging at our center, or those in whom the interval from the start of chemotherapy to the first follow-up imaging was > 3 months.
2. Patients who showed a progressive disease (PD) at the first response evaluation after chemotherapy.
3. The following patients with stable disease (SD) or partial response (PR) were excluded: (a) those in whom the pancreatic tumor size was < 15 mm; (b) those who underwent only one imaging follow-up evaluation, or whose serial assessments from baseline to study endpoint were performed using magnetic resonance imaging (MRI), or who presented with incomplete computed tomography (CT) image series; (c) those with incomplete documentation of clinical therapeutic interventions.

## CT image acquisition

For patients in center 1, the baseline and post-chemotherapy abdominal CT examinations were performed using a 64-detector spiral CT system (Discovery CT750 HD, GE System, Milwaukee, WI, USA), a 192-detector spiral CT system (SOMATOM Force CT, Siemens Healthcare, Erlangen, Germany), a 128-detector spiral CT system (Spectral CT 7500, Philips System, Cleveland, OH, USA), or a 80-detector spiral CT system (uCT 780, United Imaging Healthcare, Shanghai, China). The acquisition parameters were as follows: 120 kVp, 150‒300 mA of automatic adjustment, slice thickness of 5 mm, and pitch of 0.984:1. Contrast-enhanced CT images were obtained after a bolus intravenous injection of 1.5 ml/kg nonionic contrast agent (Ultravist 370; Bayer Healthcare, Guangzhou, China; Ioversol 350, Jiangsu Hengrui Pharmaceuticals Co., Ltd., Jiangsu, China; or Iomeprol 350, Bracco Imaging China, Shanghai, China) through an antecubital vein, at a rate of 2.5‒3 ml/s. Multiphase images were obtained at 35‒45 s (arterial phase), 65‒70 s (venous phase), and 180 s (delayed phase). The CT examinations were reconstructed using Standard (soft) kernel. These CT scan parameters were consistent with those in our previous study^1,2^.

The external test cohort (center 2) performed the examinations using a 64-detector spiral CT system (Discovery CT750 HD, GE System, Milwaukee, WI, USA) or a 64-detector spiral CT system (Sensation 64, Siemens Healthcare, Erlangen, Germany). The acquisition parameters were as follows: 120 kVp, 150‒300 mA of automatic adjustment, slice thickness of 5 mm, and pitch of 1.399:1‒0.984:1. Contrast-enhanced CT images were obtained after a bolus intravenous injection of 1.5 ml/kg nonionic contrast agent (Ultravist 370; Bayer Healthcare, Guangzhou, China; Ioversol 350, Jiangsu Hengrui Pharmaceuticals Co., Ltd., Jiangsu, China) through an antecubital vein, at a rate of 2.5‒3 ml/s. Multiphase images were obtained at 35‒45 s (arterial phase), 65‒70 s (venous phase), and 180 s (delayed phase). The CT images were reconstructed using Standard (soft) kernel for the GE system and B20f kernel for the Siemens system.

## Evaluation of inter-observer agreement

Prior to consensus review, the initial independent assessments were recorded to evaluate inter-observer agreement. The agreement for chemotherapy response classification according to RECIST 1.1 was excellent, yielding a Cohen’s kappa coefficient of 0.86 (95% CI: [0.83, 0.89]). For continuous SLD (sum of the longest diameters of target lesions) measurements, inter-observer reproducibility was excellent, with an intraclass correlation coefficient (ICC) of 0.899 (95% CI: [0.89, 0.91]).

## Volume-of-interest segmentation

The three-dimensional volume of interest (VOI) of the pancreatic tumor was manually delineated by a radiologist with 10 years of subspecialty experience in pancreatic oncology, utilizing the open-source software ITK-SNAP (version 3.8.0, [www.itksnap.org](http://www.itksnap.org/)). VOIs were annotated along tumor margins on each transverse section (slice thickness 5 mm) until complete tumor coverage was achieved. Peripheral vessels or fused lymph nodes were avoided, and necrosis within the tumor was included. All the triphasic enhanced images were analyzed, and VOIs were delineated independently for each image of the three phases. The pancreatic tumor segmentation protocol employed by this radiologist is consistent with our previous study^1,2^. For inter-observer reproducibility assessment, an independent second radiologist (with 5 years of experience) re-segmented the venous-phase VOIs, blinded to the original annotations, for a randomly selected subset of 35 patients from the training cohort. Intra-observer agreement was evaluated using the Dice similarity coefficient (DSC), yielding a mean DSC of 0.9203 ± 0.0856.

## CT image preprocessing

As convolutional neural networks (CNNs) operate on pixel intensity values, minimizing inter-image intensity variability is crucial for subsequent analyses. To mitigate the impact of different CT acquisition parameters across centers and scanners, four preprocessing steps were applied to before feature extraction: (1) spatial normalization, (2) intensity normalization, (3) region-of-interest (ROI) cropping, and (4) data augmentation. Spatial and intensity normalization followed the procedures in nnU-Net^3^, in which standardization of voxel spacing and intensity distributions are crucial for medical image analysis.

For spatial normalization, given the 2D modeling strategy, slice thickness was considered. To account for in-plane resolution variability, the median voxel spacing in the X and Y axes was calculated and used as the target spacing, while the Z-axis spacing was preserved. Accordingly, the in-plane resolution was resampled to 0.7421875 × 0.7421875 mm^2^ using trilinear interpolation.

For intensity normalization, foreground voxels (randomly sampled at approximately 10%) extracted to estimate the global foreground mean, standard deviation, and 0.5th and 99.5th percentile intensity values. Images were clipped using these statistics and underwent Z-score normalization to achieve consistent intensity distributions.

To reduce computational burden and focus model learning on lesion–relevant regions, a lesion-centered ROI was cropped from each CT slice and used as network input. The ROI was defined as a 2d×2d bounding box centered on the midpoint of lesion diameter measurement line on the baseline CT scan, where *d* represents the baseline lesion diameter. The same ROI size was applied to all subsequent follow-up scans.

Finally, spatial data augmentation was applied to the cropped ROIs to improve model robustness, including random rotation (-90° to 90°) along the X/Y axes with a probability of 40% and random left-right flipping with a probability of 20%.

## Deep feature extraction for pancreatic tumor

To extract discriminative deep features from pancreatic tumors, we employed a CNN based on the residual neural network architecture. The CNN was trained using all CT images (including both baseline and follow-up scans) from the training cohort to learn a lesion recognition model that distinguishes pancreatic tumor tissue from surrounding normal tissue. This design enables robust feature extraction from both intratumoral regions and lesion margins.

VOIs containing pancreatic cancer lesions were cropped into 2D slices, yielding a total of 10,465 slices as positive samples. An equal number of non-lesion slices, including normal pancreatic tissue and non-pancreatic tissues, were collected as negative controls from regions adjacent to the tumors. To prevent data leakage, all data were split at patient level into independent training (145 patients), validation (48 patients), and test sets (50 patients). The number of lesion and non- lesion slices in each set were as follows: training set, 5,937 each; validation set, 2,395 each; test set, 2,133 each. The CNN model achieving the best classification performance on the test set was selected as the final feature extractor for subsequent deep feature extraction. This model achieved an accuracy of 0.881 and an area under the curve (AUC) of 0.954.

Figure 2B illustrates the lesion and non-lesion cropping strategy. Lesion tissue patches were extracted from pancreatic tumor regions within lesion-containing slices, indicated by red bounding boxes. Non-lesion tissue patches were obtained from two sources: (1) normal pancreatic or non-pancreatic tissues within lesion-containing slices and (2) perilesional tissues from the two adjacent slices immediately above and below the lesion slice along the Z-axis, indicated by green bounding boxes. For each time point, non-lesion tissue patches were uniformly sampled from both sources to match the number of lesion tissue patches per case.

## Comparison with other methods

The transformer-based framework adopts an encoder-decoder structure. The encoder receives the 512-dimensional deep features extracted from CT images, enhanced with time positional encoding (TPE). It consists of six repetitive blocks. Each block contains a time-sensitive multi-head self-attention (TMSA) layer and a multilayer perceptron (MLP), with residual connections and layer normalization applied around each layer. The encoded image features are then forwarded to the decoder. Meanwhile, the decoder also receives embedded representations of image metrics and clinical variables, which are encoded into 512-dimensional vectors using an input embedding layer and enhanced with TPE. The decoder shares a similar structure with the encoder and also has six stacked blocks. Each block includes three layers: self-attention on decoder inputs, cross-attention between first layer outputs and encoder outputs, and an MLP layer. The decoder’s final output passes through a linear layer and softmax to predict the probability of disease progression at next follow-up visit. The two key components, TPE and TMSA, are detailed below.

(1) TPE. In the original transformer, the self-attention mechanism is permutation-invariant, and therefore positional encoding is required to capture sequence order. TPE extends the standard sinusoidal positional encoding by incorporating temporal interval information^4^:

$\mathrm{TPE}\left( \Delta t_{i},q \right)= \left\{ \begin{aligned} \sin\left( \omega_{q}\cdot\Delta t_{i,0} \right), q=2k \\ \cos(\omega_{q}\cdot\Delta t_{i,0}), q=2k+1 \end{aligned} \right.$

where $\Delta t_{i,1}$denotes the time interval between the i-th follow-up and baseline, and $\omega_{q}=1/{{10000}^{{2q}/{d_{m}}}},q\in\left[ 0,d_{m} \right].$ The modified encoding not only represents sequential order but also explicitly encodes temporal distances.

(2) TMSA. Self-attention naturally captures long-range dependencies but does not inherently account for varying temporal relationships in sequential medical data. Recent data typically exerts greater influence on predictions than distant historical data. Therefore, we incorporate temporal interval information into multi-head self-attention. Self-attention is expressed as:

$\varsigma\left( Q,K,T,V \right)=softmax(\frac{QK^{T}\circ T}{\sqrt{d_{m}}})V$

$Q= \tilde{f}W^{Q}$

$K= \tilde{f}W^{K}$

$V= \tilde{f}W^{V}$

where $\tilde{f}\in\mathbb{R}^{{n\times d}_{m}}$ represents encoder input features. The query matrix$Q \in\mathbb{R}^{n\times d_{q}}$, key matrix $K\in\mathbb{R}^{n\times d_{k}}$, and value matrix $V\in\mathbb{R}^{n\times d_{v}}$ are obtained by multiplying $\tilde{f}$ with three trainable matrices $W^{Q}$，$W^{K}$，and $W^{V}$. The dimensions are set as $d_{q}{=d}_{k}=d_{v}=d_{m}=512$. $T\in\mathbb{R}^{d_{m}\times d_{m}}$ is a weight matrix related to temporal interval, and $\circ$ denotes the Hadamard (element-wise) product.

Let $P=QK^{T}$ where $P\in\mathbb{R}^{n\times n}$. Element $P_{i,j}$ represents the influence of follow-up j on follow-up i. For temporal data, especially long sequences, recent data deserves greater attention as it reflects current treatment response—crucial for predicting future progression. The element-wise multiplication of matrix P with temporal interval matrix T encourages the model to focus more on recent time intervals. Each element of T is computed as:

$$T_{i,j}=\frac{1}{e^{A\Delta t_{i,j}+B}}$$

where A and B are learnable parameters. Dividing $P\circ T$ by $\sqrt{d_{m}}$ stabilizes gradient propagation before the softmax operation. Following the multi-head approach in transformer, we use multiple attention heads with $Q_{i}\in\mathbb{R}^{n\times d_{q}/s}$, $K_{i}\in\mathbb{R}^{n\times d_{k}/s}$, $V_{i}\in\mathbb{R}^{n\times d_{v}/s}$ performing s self-attention operations. The s outputs are concatenated and linearly projected to dimension $d_{m}$

$$\mathcal{H}\left( Q,K,V,T \right)=Concat(H_{1},\ldots H_{s})W^{O}$$

where $H_{i}=\varsigma(Q_{i}, K_{i}, V_{i}, T_{i})$, and $W^{O}$ is a projection matrix. We set $s=8$, $d_{v}={d_{m}}/s=64$.

## Experimental setup

For training the lesion classification model (step 2 in Figure 2B), the Adam optimizer was employed with an initial learning rate of 5e-3. The learning rate was dynamically adjusted using a cosine annealing scheduler, gradually decreasing from the initial value over 120 epochs. This strategy mitigates gradient oscillations and enhances training stability by allowing finer parameter updates near the loss minimum in later stages. Cross-entropy loss was used to quantify the discrepancy between model predictions and ground-truth labels.

For training the progression prediction model (step 3 in Figure 2B), the Adam optimizer was also used with an initial learning rate of 1e-3. The learning rate was similarly adjusted using cosine annealing over 100 epochs, and cross-entropy loss was applied as the optimization objective. All experiments were conducted on a server equipped with two NVIDIA RTX A6000 GPUs, using Python 3.8.13 and PyTorch 1.13.

## References

1. Yang Q, Mao Y, Han Y, et al: Noninvasive Computed Tomography-Based Quantification of Tumor Fibrosis Predicts Pancreatic Cancer Response to Gemcitabine/Nab-Paclitaxel. Research (Wash D C) 8:0937, 2025

2. Yang Q, Mao Y, Xie H, et al: Identifying Outcomes of Patients With Advanced Pancreatic Adenocarcinoma and RECIST Stable Disease Using Radiomics Analysis. JCO Precision Oncology 6:e2100362, 2022

3. Isensee F, Petersen J, Klein A, et al: Abstract: nnU-Net: Self-adapting Framework for U-Net-Based Medical Image Segmentation. Bildverarbeitung für die Medizin 2019. Wiesbaden, Springer Fachmedien Wiesbaden, 2019, pp 22-22

4. Hu X, Zhang LX, Gao L, et al: GLIM-Net: Chronic Glaucoma Forecast Transformer for Irregularly Sampled Sequential Fundus Images. IEEE Trans Med Imaging 42:1875-1884, 2023

# Supplementary Tables

**Supplementary Table 1.** Baseline hematological parameters of patients.

| **Parameter** | **Training cohort, N=243** | **Internal test, N=41** | **External test, N=46** | **Prospective cohort, N=57** | **P value** |
| --- | --- | --- | --- | --- | --- |
| Neutrophil count  Median (range), 10⁹/L | 4.45  (0.93, 13.88) | 5.09  (1.75, 10.5) | 4.85  (1.41, 10.55) | 4.63  (2.36, 11.75) | 0.291 |
| Neutrophil percentage  Median (range), % | 68.0  (39, 90.7) | 68.3  (45.4, 80.3) | 67.4  (37.1, 83.7) | 67.6  (45.1, 81.2) | 0.981 |
| Lymphocyte count  Median (range), 10⁹/L | 1.47  (0.57, 3.58) | 1.7  (0.54, 3.07) | 1.47  (0.6, 3.33) | 1.71  (0.75, 2.94) | 0.024 |
| Lymphocyte percentage  Median (range), % | 22.3  (3.7, 47.5) | 22.7  (9.8, 41.9) | 23.4  (9.2, 39.3) | 21.7  (10.1, 45.6) | 0.992 |
| Monocyte count  Median (range), 10⁹/L | 0.45  (0.1, 1.45) | 0.51  (0.1, 1.1) | 0.41  (0.18, 1.25) | 0.49  (0.2, 1.22) | 0.163 |
| Monocyte percentage  Median (range), % | 6.7  (2.4, 13.8) | 7.0  (4.2, 15.0) | 6.6  (3.9, 33) | 6.7  (2.2, 12.1) | 0.746 |
| Hemoglobin  Median (range), g/L | 132  (83, 170) | 128  (91, 160) | 127  (93, 156) | 132  (104, 166) | 0.112 |
| Albumin  Median (range), g/L | 43.8  (23.1, 54.7) | 43.5  (31.6, 53.3) | 39.4  (30.7, 48.2) | 44.6  (29.7, 53.5) | <0.001 |

**Supplementary Table 2.** Predictive performance of cross-validation derived models across cohorts.

| **Dataset** | **Model** | **AUC** | **Accuracy** | **Sensitivity** | **Specificity** | **F1 score** |
| --- | --- | --- | --- | --- | --- | --- |
| Training | Fold-1 | 0.71 | 0.69 | 0.82 | 0.65 | 0.66 |
|  | Fold-2 | 0.71 | 0.73 | 0.52 | 0.85 | 0.69 |
|  | Fold-3 | 0.83 | 0.81 | 0.83 | 0.80 | 0.78 |
|  | Fold-4 | 0.83 | 0.75 | 0.85 | 0.71 | 0.74 |
|  | Fold-5 | 0.80 | 0.85 | 0.68 | 0.91 | 0.81 |
|  | Mean±SD (95% CI) | 0.78±0.06  (0.70, 0.85) | 0.77±0.06  (0.69, 0.85) | 0.74±0.14  (0.57, 0.91) | 0.78±0.14  (0.65, 0.91) | 0.74±0.06  (0.66, 0.81) |
| Internal test | Fold-1 | 0.75 | 0.77 | 0.75 | 0.78 | 0.72 |
|  | Fold-2 | 0.77 | 0.72 | 0.81 | 0.69 | 0.68 |
|  | Fold-3 | 0.75 | 0.70 | 0.75 | 0.69 | 0.66 |
|  | Fold-4 | 0.75 | 0.73 | 0.69 | 0.75 | 0.67 |
|  | Fold-5 | 0.74 | 0.73 | 0.88 | 0.69 | 0.70 |
|  | Mean±SD (95% CI) | 0.75±0.01  (0.74, 0.77) | 0.73±0.03  (0.70, 0.76) | 0.78±0.07  (0.69, 0.87) | 0.72±0.04  (0.67, 0.77) | 0.69±0.02  (0.66, 0.72) |
| External test | Fold-1 | 0.72 | 0.60 | 0.92 | 0.45 | 0.59 |
|  | Fold-2 | 0.74 | 0.73 | 0.80 | 0.70 | 0.71 |
|  | Fold-3 | 0.73 | 0.78 | 0.60 | 0.86 | 0.73 |
|  | Fold-4 | 0.74 | 0.72 | 0.76 | 0.70 | 0.70 |
|  | Fold-5 | 0.78 | 0.74 | 0.72 | 0.75 | 0.72 |
|  | Mean±SD (95% CI) | 0.74±0.02  (0.72, 0.77) | 0.71±0.07  (0.62, 0.80) | 0.76±0.12  (0.61, 0.91) | 0.69±0.15  (0.50, 0.88) | 0.69±0.06  (0.62, 0.76) |

**Supplementary Table 3.** Predictive performance of cross-validation derived models constructed with different feature combinations. Dim, dimension.

| **Dataset** | **Dim** | **AUC**  mean (95% CI) | **Accuracy**  mean (95% CI) | **Sensitivity**  mean (95% CI) | **Specificity**  mean (95% CI) |
| --- | --- | --- | --- | --- | --- |
| Training | 512 | 0.68  (0.60, 0.76) | 0.77  (0.72, 0.82) | 0.52  (0.38, 0.67) | 0.88  (0.80, 0.95) |
|  | 515 | 0.76  (0.70, 0.82) | 0.76  (0.70, 0.82) | 0.65  (0.56, 0.74) | 0.81  (0.75, 0.86) |
|  | 516 | 0.77  (0.70, 0.84) | 0.75  (0.65, 0.85) | 0.72  (0.60, 0.84) | 0.77  (0.59, 0.95) |
|  | **529** | **0.78**  **(0.70, 0.85)** | **0.77**  **(0.69, 0.85)** | **0.74**  **(0.57, 0.91)** | **0.78**  **(0.65, 0.91)** |
| Internal test | 512 | 0.71  (0.68, 0.73) | 0.87  (0.85, 0.89) | 0.55  (0.52, 0.58) | 0.96  (0.94, 0.98) |
|  | 515 | 0.70  (0.66, 0.74) | 0.75  (0.68, 0.82) | 0.65  (0.55, 0.75) | 0.78  (0.68, 0.89) |
|  | 516 | 0.72  (0.67, 0.76) | 0.68  (0.64, 0.73) | 0.79  (0.70, 0.88) | 0.65  (0.58, 0.72) |
|  | **529** | **0.75**  **(0.74, 0.77)** | **0.73**  **(0.70, 0.76)** | **0.78**  **(0.69, 0.87)** | **0.72**  **(0.67, 0.77)** |
| External test | 512 | 0.65  (0.63, 0.67) | 0.69  (0.63, 0.75) | 0.55  (0.36, 0.74) | 0.75  (0.58, 0.92) |
|  | 515 | 0.62  (0.58, 0.66) | 0.72  (0.68, 0.76) | 0.46  (0.31, 0.60) | 0.83  (0.74, 0.93) |
|  | 516 | 0.66  (0.63, 0.69) | 0.71  (0.62, 0.80) | 0.46  (0.25, 0.67) | 0.82  (0.60, 1.0) |
|  | **529** | **0.74**  **(0.72, 0.77)** | **0.71**  **(0.62, 0.80)** | **0.76**  **(0.61, 0.91)** | **0.69**  **(0.50, 0.88)** |

**Supplementary Table 4.** Subgroup analysis of predictive performance by chemotherapy regimen.

| **Chemotherapy regimen** | **AUC** | **Accuracy** | **Sensitivity** | **Specificity** | **P-value** |
| --- | --- | --- | --- | --- | --- |
| AG or gemcitabine-based, N=168 (n=304) | 0.79 | 0.78 | 0.76 | 0.78 | reference |
| FOLFIRINOX, N=73 (n=108) | 0.68 | 0.69 | 0.55 | 0.75 | 0.11 |
| SOXIRI, N=43 (n=74) | 0.77 | 0.74 | 0.60 | 0.80 | 0.77 |

Note: N is the number of patients, and n is the number of prediction events.

**Supplementary Table 5.** Subgroup analysis of predictive performance by progression type.

| **Progression type** | **AUC** | **Accuracy** | **Sensitivity** | **Specificity** | **P-value** |
| --- | --- | --- | --- | --- | --- |
| Type 1, N=84 (n=160) | 0.72 | 0.71 | 0.69 | 0.74 | 0.51 |
| Type 2, N=54 (n=106) | 0.77 | 0.74 | 0.70 | 0.80 |  |

Note: N is the number of patients, and n is the number of prediction events. Unlike other subgroup analyses, the total number of patients in this table (N=138) comprises only those who experienced progressive disease during the study period.

**Supplementary Table 6.** Subgroup analysis of predictive performance by baseline disease stage.

| **Baseline disease stage** | **AUC** | **Accuracy** | **Sensitivity** | **Specificity** | **P-value** |
| --- | --- | --- | --- | --- | --- |
| Non-metastatic, N=90 (n=144) | 0.85 | 0.87 | 0.75 | 0.89 | 0.0363 |
| Metastatic, N=194 (n=342) | 0.71 | 0.70 | 0.68 | 0.71 |  |

Note: N is the number of patients, and n is the number of prediction events.

**Supplementary Table 7.** Subgroup analysis of predictive performance by tumor location.

| **Tumor location** | **AUC** | **Accuracy** | **Sensitivity** | **Specificity** | **P-value** |
| --- | --- | --- | --- | --- | --- |
| Head and neck, N=97 (n=157) | 0.73 | 0.75 | 0.61 | 0.80 | 0.38 |
| Body and tail, N=187 (n=329) | 0.78 | 0.75 | 0.73 | 0.76 |  |

Note: N is the number of patients, and n is the number of prediction events.

**Supplementary Table 8.** Subgroup analysis of predictive performance by longitudinal sequence length

| **Sequence length** | **AUC** | **Accuracy** | **Sensitivity** | **Specificity** | **P-value** |
| --- | --- | --- | --- | --- | --- |
| $\leq2$, N=247 | 0.73 | 0.72 | 0.62 | 0.79 | 0.91 |
| $>2$, N=37 | 0.71 | 0.89 | 0.97 | 0.57 |  |

Note: Predictive performance was evaluated exclusively at the patient's final follow-up time point. Therefore, the number of prediction events equals the number of patients (N).

**Supplementary Table 9.** Performance comparison between different methods.

| **Dataset** | **Method** | **AUC** | **Accuracy** | **Sensitivity** | **Specificity** |
| --- | --- | --- | --- | --- | --- |
| Training | Single time point, SVM | 0.49 | 0.71 | 0.01 | 1 |
|  | Single time point, LR | 0.62 | 0.70 | 0.22 | 0.90 |
|  | Sequence, transformer | 0.68 | 0.72 | 0.64 | 0.73 |
|  | **Ours (LSTM)** | **0.78** | **0.77** | **0.74** | **0.78** |
| Internal test | Single time point, SVM | 0.49 | 0.77 | 0 | 1 |
|  | Single time point, LR | 0.50 | 0.71 | 0.11 | 0.89 |
|  | Sequence, transformer | 0.79 | 0.70 | 0.87 | 0.64 |
|  | **Ours (LSTM)** | **0.75** | **0.73** | **0.78** | **0.72** |
| External test | Single time point, SVM | 0.51 | 0.69 | 0 | 1 |
|  | Single time point, LR | 0.58 | 0.67 | 0.04 | 0.95 |
|  | Sequence, transformer | 0.67 | 0.69 | 0.70 | 0.68 |
|  | **Ours (LSTM)** | **0.74** | **0.71** | **0.76** | **0.69** |

Note: Our method was compared with machine learning models built on single-time-point data and a transformer-based model built on sequential data.

SVM, support vector machine; LR, logistic regression; LSTM, long short-term memory.

**Supplementary Table 10.** Ablation study of the TPE and TMSA modules in the transformer-based method.

| **Dataset** | **TPE** | **TMSA** | **AUC** | **Accuracy** | **Sensitivity** | **Specificity** |
| --- | --- | --- | --- | --- | --- | --- |
| Training | × | × | 0.63 | 0.70 | 0.54 | 0.77 |
|  | √ | × | 0.67 | 0.70 | 0.62 | 0.73 |
|  | √ | √ | 0.68 | 0.72 | 0.64 | 0.73 |
| Internal test | × | × | 0.77 | 0.77 | 0.71 | 0.78 |
|  | √ | × | 0.78 | 0.70 | 0.85 | 0.66 |
|  | √ | √ | 0.79 | 0.70 | 0.87 | 0.64 |
| External test | × | × | 0.64 | 0.68 | 0.60 | 0.72 |
|  | √ | × | 0.66 | 0.68 | 0.69 | 0.67 |
|  | √ | √ | 0.67 | 0.69 | 0.70 | 0.68 |

TPE, time positional encoding; TMSA, time-sensitive multi-head self-attention.

**Supplementary Table 11.** Comparison of model predictive performance using different feature scaling strategies.

| **Strategy** | **AUC** | **Accuracy** | **Sensitivity** | **Specificity** |
| --- | --- | --- | --- | --- |
| Original Scale (Adopted) | 0.78 | 0.77 | 0.74 | 0.78 |
| Min-Max Normalization | 0.75 | 0.73 | 0.68 | 0.75 |
| Z-score Standardization | 0.75 | 0.71 | 0.68 | 0.73 |
| Log + Standardization: | 0.76 | 0.71 | 0.65 | 0.73 |

**Note:** Results represent average metrics from 5-fold cross-validation. "Log + Standardization" indicates applying a logarithmic transformation followed by Z-score standardization.

**Supplementary Table 12.** Event labels and sequence lengths in the training cohort.

| **Training cohort** | **Baseline** | **CT 1** | **Follow-ups before study endpoint** | | | | | | | **Patients**  **N=243** |
| --- | --- | --- | --- | --- | --- | --- | --- | --- | --- | --- |
|  |  |  | **CT 2** | **CT 3** | **CT 4** | **CT 5** | **CT 6** | **CT 7** | **CT 8** |  |
| **Label** 1 | √ | √ | 55 PD /  188 non-PD |  |  |  |  |  |  | 118 |
| **Label** 2 | √ | √ | √ | 42 PD /  83 non-PD |  |  |  |  |  | 95 |
| **Label** 3 | √ | √ | √ | √ | 16 PD /  14 non-PD |  |  |  |  | 20 |
| **Label** 4 | √ | √ | √ | √ | √ | 5 PD /  5 non-PD |  |  |  | 5 |
| **Label** 5 | √ | √ | √ | √ | √ | √ | 3 PD /  2 non-PD |  |  | 4 |
| **Label** 6 | √ | √ | √ | √ | √ | √ | √ | 0 /  1 non-PD |  | 0 |
| **Label** 7 | √ | √ | √ | √ | √ | √ | √ | √ | 1 PD /0 | 1 |
| **Events,** n=415  (122 PD/ 293 non-PD) |  |  | 243 | 125 | 30 | 10 | 5 | 1 | 1 |  |

**Supplementary Table 13.** Event labels and sequence lengths in the internal test cohort.

| **Internal test cohort** | **Baseline** | **CT 1** | **Follow-ups before study endpoint** | | | | **Patients**  **N=41** |
| --- | --- | --- | --- | --- | --- | --- | --- |
|  |  |  | **CT 2** | **CT 3** | **CT 4** | **CT 5** |  |
| **Label** 1 | √ | √ | 3 PD /  38 non-PD |  |  |  | 19 |
| **Label** 2 | √ | √ | √ | 8 PD /  14 non-PD |  |  | 15 |
| **Label** 3 | √ | √ | √ | √ | 4 PD /  3 non-PD |  | 6 |
| **Label** 4 | √ | √ | √ | √ | √ | 1 PD /0 | 1 |
| **Events,** n=71  (16 PD/ 55 non-PD) |  |  | 41 | 22 | 7 | 1 |  |

**Supplementary Table 14.** Event labels and sequence lengths in the external test cohort.

| **External test cohort** | **Baseline** | **CT 1** | **Follow-ups before study endpoint** | | | | **Patients**  **N=46** |
| --- | --- | --- | --- | --- | --- | --- | --- |
|  |  |  | **CT 2** | **CT 3** | **CT 4** | **CT 5** |  |
| **Label** 1 | √ | √ | 9 PD /  37 non-PD |  |  |  | 22 |
| **Label** 2 | √ | √ | √ | 9 PD /  15 non-PD |  |  | 14 |
| **Label** 3 | √ | √ | √ | √ | 7 PD /  3 non-PD |  | 9 |
| **Label** 4 | √ | √ | √ | √ | √ | 0 /  1 non-PD | 1 |
| **Events,** n=81  (25 PD/ 56 non-PD) |  |  | 46 | 24 | 10 | 1 |  |

**Supplementary Table 15.** Event labels and sequence lengths in the prospective cohort.

| **Prospective cohort** | **Baseline** | **CT 1** | **Follow-ups before study endpoint** | | | | **Patients**  **N=57** |
| --- | --- | --- | --- | --- | --- | --- | --- |
|  |  |  | **CT 2** | **CT 3** | **CT 4** | **CT 5** |  |
| **Label** 1 | √ | √ | 9 PD /  48 non-PD |  |  |  | 39 |
| **Label** 2 | √ | √ | √ | 6 PD /  12 non-PD |  |  | 15 |
| **Label** 3 | √ | √ | √ | √ | 1 PD /  2 non-PD |  | 2 |
| **Label** 4 | √ | √ | √ | √ | √ | 1 PD /0 | 1 |
| **Events,** n= 79  (17 PD/ 62 non-PD) |  |  | 57 | 18 | 3 | 1 |  |

# Supplementary legend for Figure 5

**Patient 1:** A 71-year-old female presented with pancreatic ductal adenocarcinoma in the head/neck of the pancreas. At 2.5 months post-chemotherapy, the pancreatic mass demonstrated significant regression, assessed as a partial response (PR) per RECIST 1.1, with a short-term progressive disease (PD) probability of 0.092 predicted by the proposed model. At 5.3 months, further reduction of the pancreatic lesion was observed (PR), yet the model predicted a PD probability of 0.997. By 7.9 months, while the pancreatic lesion remained stable, new peritoneal metastases and a small amount of ascites were identified, confirming PD.

**Patient 2:** A 38-year-old female presented with a pancreatic body mass and multiple hepatic metastases. At 1.5 months post-chemotherapy, significant regression of both the pancreatic mass and hepatic metastases was observed, assessed as PR per RECIST 1.1, with a short-term PD probability of 0.012 predicted by the proposed model. At 3.0 months, continued reduction of pancreatic and hepatic lesions was documented (PR), with a model-predicted PD probability of 0.009. By 5.7 months, though clinically assessed as PR, the pancreatic lesion showed slight enlargement with concurrent growth of one metastasis in hepatic segment S5, while the model predicted a PD probability of 0.991. At 6.7 months, progression was confirmed (PD) with enlargement of the pancreatic lesion, marked enlargement of one S5 metastasis, and slight enlargement of another S5 metastasis.

# Supplementary Figures

**
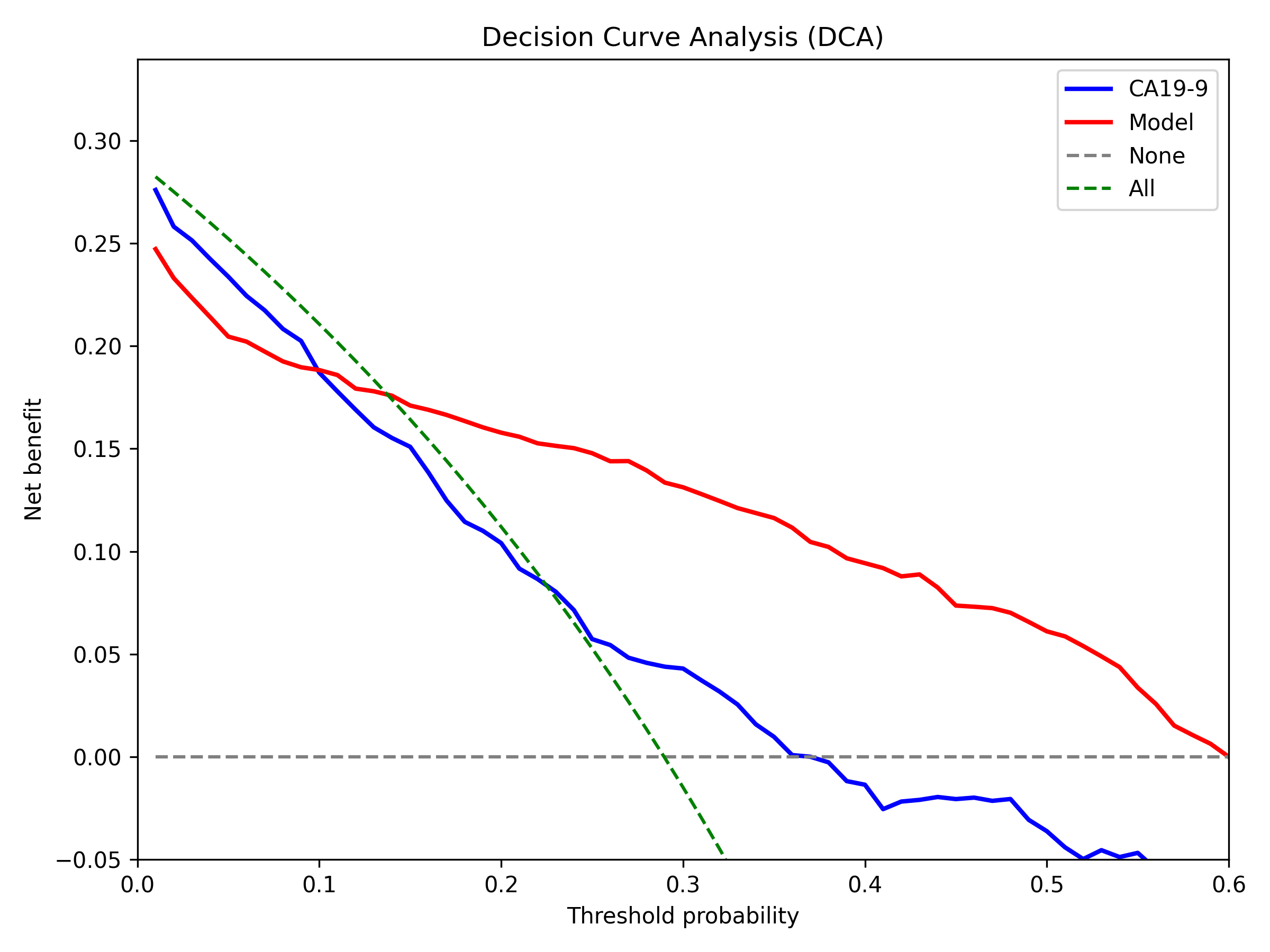
**

**Supplementary Figure 1.** Decision curve analysis comparing the clinical utility of the proposed model (red line) and CA19-9 (blue line).


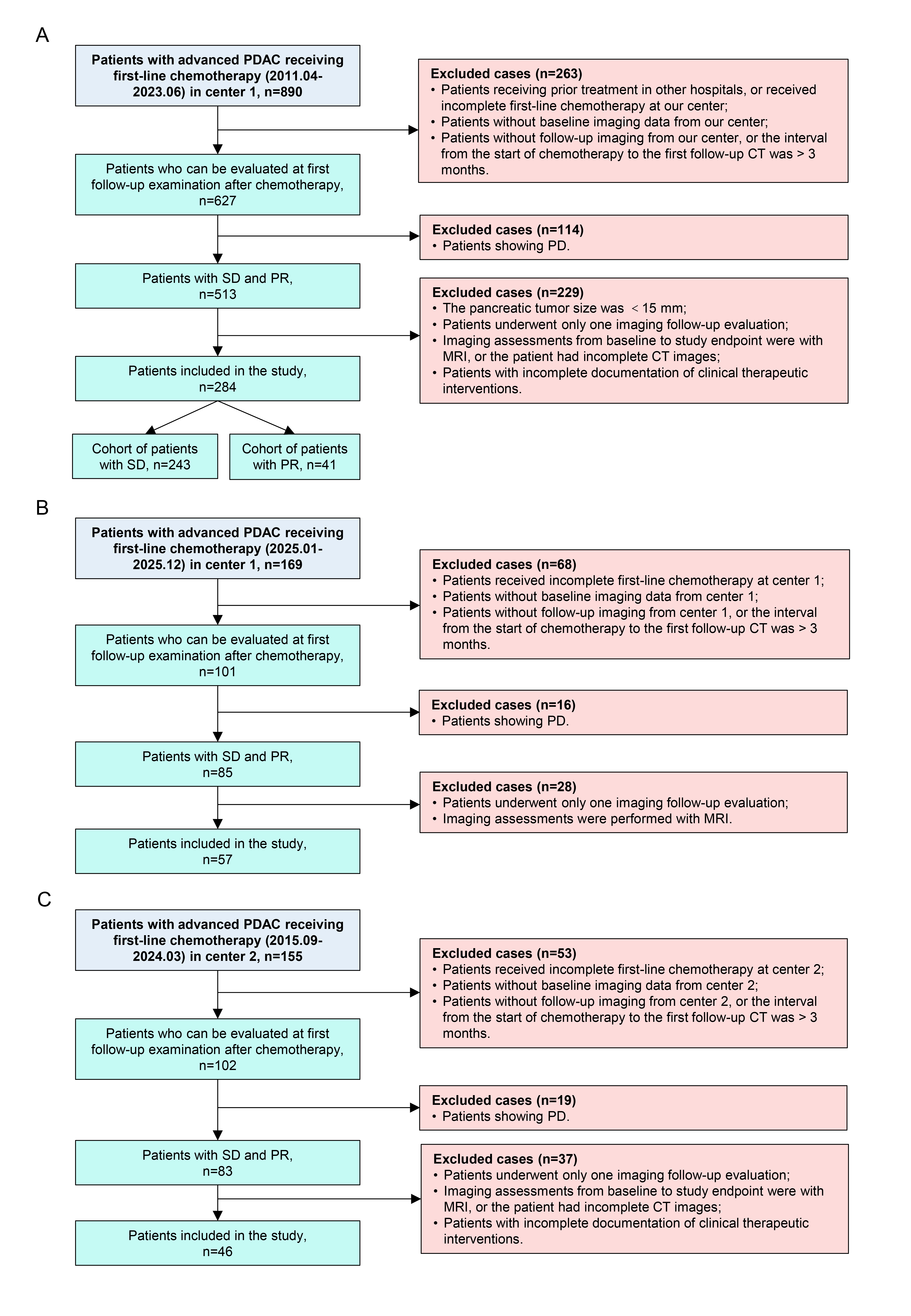


**Supplementary Figure 2.** Patient enrollment flowchart.

Abbreviation: PDAC, Pancreatic Ductal Adenocarcinoma; SD, Stable Disease; PR, Partial Response; PD, Progressive Disease.


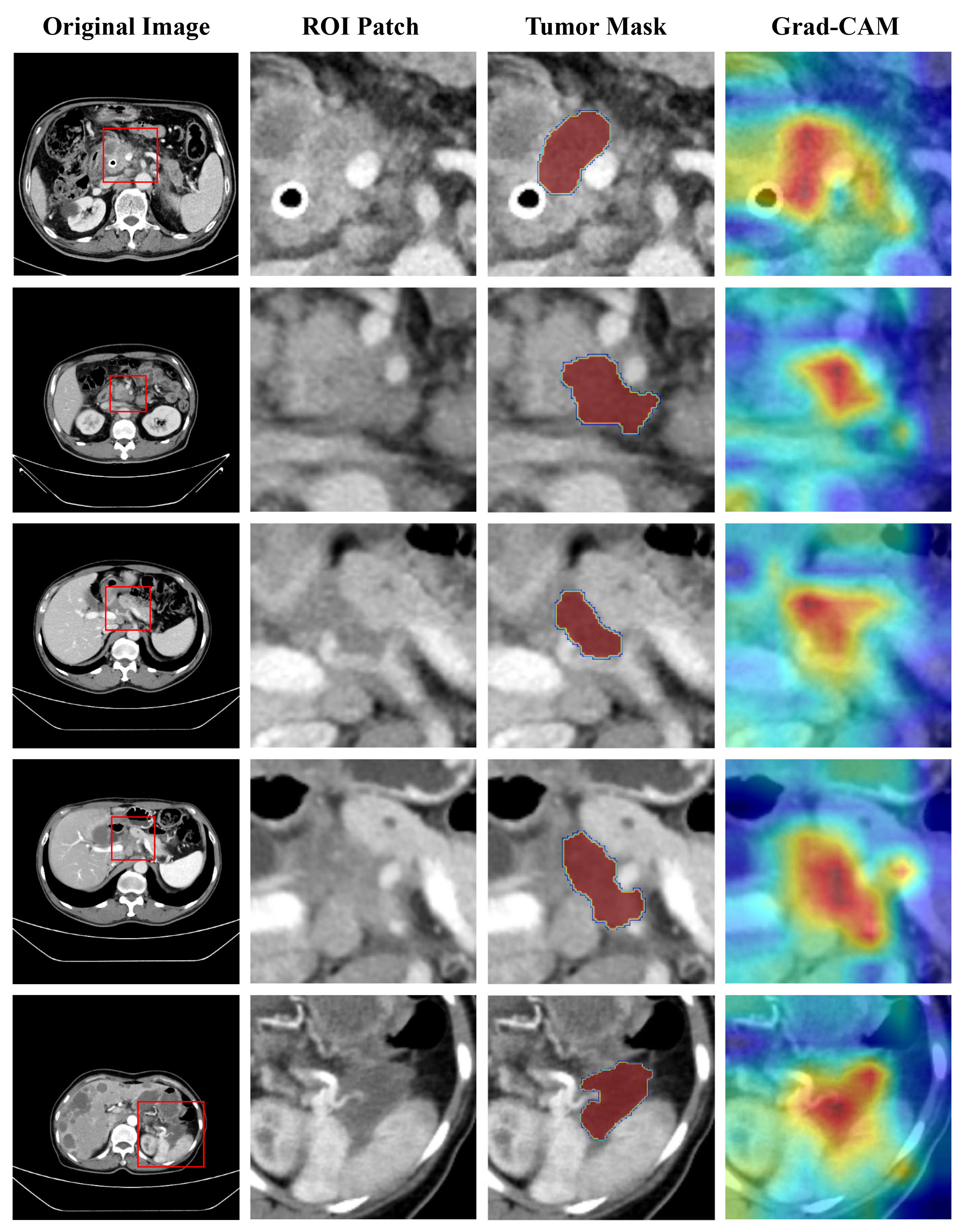


**Supplementary Figure 3.** Gradient-weighted class activation mapping (Grad-CAM) visualization of lesion-focused feature extraction. Representative CT images, cropped ROI patches, annotated tumor masks, and corresponding Grad-CAM heatmaps are shown. High-response regions (red-yellow) largely overlap with the annotated tumor areas, indicating that the feature extractor predominantly attends to clinically relevant lesion regions.


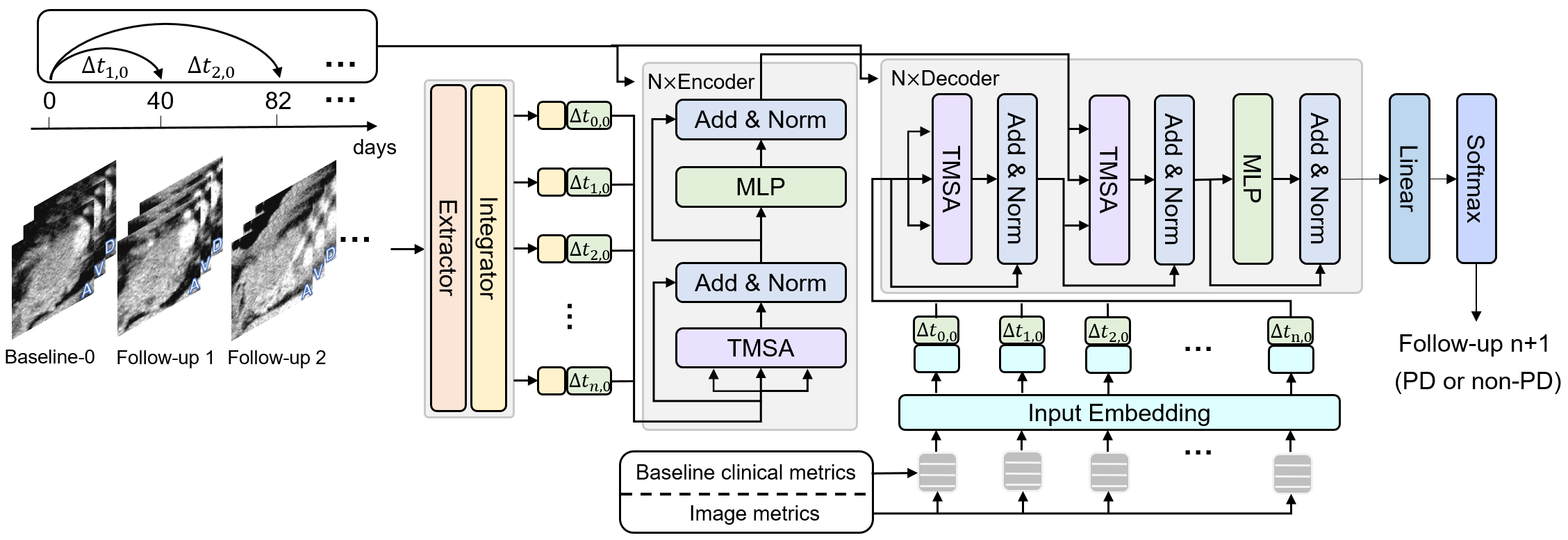


**Supplementary Figure 4.** Transformer-based deep learning framework for predicting disease progression.
